# Acetylation as a dynamic regulatory interface between plant stress memory, cross-tolerance, and crop resilience design

**DOI:** 10.3389/fpls.2026.1869320

**Published:** 2026-06-17

**Authors:** Rongjin Ma, Xianjue Ruan, Xinchen Niu, Qingyuan Li, Jing Wen, Yu Pan, Chunyu Shang

**Affiliations:** College of Horticulture and Landscape Architecture, Southwest University, Chongqing, China

**Keywords:** acetylation, stress memory, cross-tolerance, crop stress resilience, epigenetic-state design, HATs, HDACs

## Abstract

Plants are increasingly exposed to recurrent, combined, and fluctuating environmental stresses, making it essential to understand how transient stress signals are converted into durable adaptive states. Stress memory and cross-tolerance represent two interconnected strategies that enable plants to respond more rapidly or effectively to subsequent stresses, yet the regulatory mechanisms linking short-term responses with long-term adaptive potential remain incompletely understood. Acetylation has emerged as a dynamic regulatory interface in this process owing to its reversibility, rapid responsiveness, broad substrate range, and close coupling with cellular metabolic status. In this review, we summarize recent progress in acetylation-mediated plant stress adaptation, focusing on transcriptional bookmarking at the chromatin level, non-histone acetylation of signaling and metabolic proteins, acetyl-CoA-dependent and NAD^+^-dependent metabolic coupling, and regulation in different subcellular compartments, including chloroplasts, mitochondria, and the cytoplasm. We further discuss the conservation, divergence, and evidence hierarchy of key HAT/HDAC regulatory nodes in model plants and crops, highlighting that their functional outputs depend on target identity, stress context, and crop background rather than on their enzymatic identity alone. Finally, we evaluate the potential and limitations of acetylation-based crop strategies for improving crop stress resilience, including priority target selection, small-molecule regulation, epigenome editing, synthetic regulatory modules, and molecular design breeding. Overall, acetylation should not be viewed simply as a transcriptional “on/off” switch, but as a multilayered regulatory hub linking environmental signals, metabolic states, chromatin plasticity, and adaptive phenotypes. Future advances will depend on causal validation of non-histone substrates, time-resolved acetylation maps, multi-stress network dissection, and validation in crop systems under complex field conditions.

## 1 Introduction

Plants are increasingly exposed to recurrent, combined, and fluctuating environmental stresses, including drought, salinity, heat, cold, high light, and pathogen infection (Zandalinas et al., 2024; Jiang et al., 2025). Under such conditions, plant stress adaptation cannot be fully explained by transient responses to individual stress events. Instead, plants often retain information from previous stress exposure and use this preconditioned state to respond more rapidly or effectively to subsequent challenges (Hilker et al., 2016; Liu et al., 2022). This capacity is closely associated with two interrelated phenomena: stress memory and cross-tolerance (Walter et al., 2013; Nair et al., 2022).

**Figure 1:**
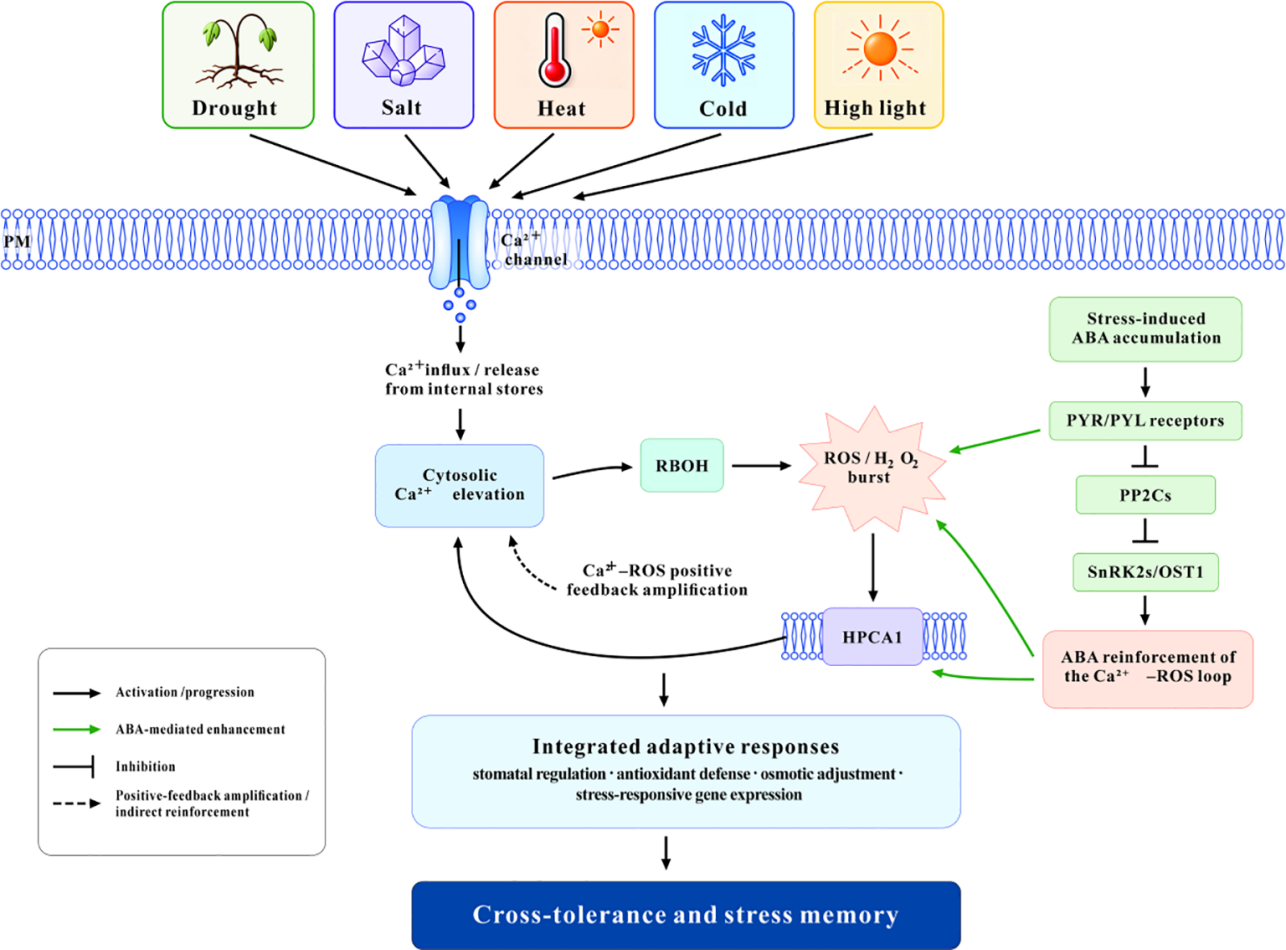
Shared Ca²^+^-ROS-ABA signaling provides a basis for plant cross-tolerance and stress-memory priming.

Stress memory refers to the ability of plants to maintain physiological, transcriptional, epigenetic, or metabolic traces after an initial stress exposure, thereby enabling faster or stronger responses upon re-exposure to the same or a related stress (Ding et al., 2012; Lämke and Bäurle, 2017; Liu et al., 2022; Charng et al., 2023). Cross-tolerance, in contrast, describes the phenomenon whereby prior exposure to one stress enhances tolerance to a different stress, usually through partially shared signaling and defense networks (Walter et al., 2013; Nair et al., 2022). Although these two processes are not identical, they are mechanistically connected: stress memory provides a temporal basis for retaining stress-induced regulatory states, whereas cross-tolerance represents a functional outcome that can arise when such states are mobilized through common signaling modules such as reactive oxygen species (ROS), Ca²^+^, and hormone-related pathways. These shared signaling modules, particularly the Ca²^+^-ROS-ABA regulatory loop, allow different stresses to converge early and trigger priming, cross-tolerance, and memory-related responses (**Figure 1**; Fichman and Mittler, 2021; Waadt et al., 2022).

Drought, salinity, heat, cold, and high light can converge on early Ca²^+^ influx and ROS/H_2_O_2_ production. An increase in cytosolic Ca²^+^ can stimulate RBOH-dependent ROS production, whereas ROS/H_2_O_2_ can further promote Ca²^+^ influx through H_2_O_2_-sensing mechanisms such as HPCA1, forming a self-amplifying Ca²^+^-ROS feedback loop (Wu et al., 2020; Fichman and Mittler, 2021). Stress-induced ABA accumulation can feed into this loop through the PYR/PYL-PP2C-SnRK2/OST1 signaling module. In particular, OST1/SnRK2.6 regulates RBOHD/RBOHF-mediated ROS production, thereby coupling ABA signaling with Ca²^+^-ROS amplification (Kwak et al., 2003; Sirichandra et al., 2009; Waadt et al., 2022). Through this loop, ABA, ROS, and Ca²^+^ signals regulate guard-cell ion channels and stomatal closure. They also promote antioxidant defense, osmotic adjustment, and stress-responsive gene expression through ROS-dependent, Ca²^+^-dependent, and ABA-related transcriptional regulation (Pei et al., 2000; Huang et al., 2019; Waadt et al., 2022). Thus, the Ca²^+^-ROS-ABA loop allows diverse stress signals to converge at an early stage and supports stress priming, cross-tolerance, and memory-related adaptive responses.

A central unresolved question is how transient stress signals are converted into regulatory states that are sufficiently stable to support memory, yet sufficiently flexible to allow resetting and reactivation (Friedrich et al., 2021; Charng et al., 2023). Early Ca^2+^, ROS, and ABA signals are rapid and transient. They explain early stress-signal convergence but not the retention or rapid reactivation of regulatory states after stress release. This conversion often requires regulatory layers that continue to influence gene expression, protein function, or metabolic status after the initial signals have declined. Epigenetic regulation and post-translational modifications therefore provide an important mechanistic bridge between short-lived stress signals and more persistent, reactivatable adaptive states.

This figure illustrates how multiple environmental stresses converge at the early Ca²^+^, ROS/H_2_O_2_, and ABA signaling levels. The Ca²^+^-ROS positive-feedback loop and the ABA-mediated PYR/PYL-PP2C-SnRK2/OST1 module jointly connect early stress perception with downstream adaptive outputs, including stomatal regulation, antioxidant defense, osmotic adjustment, and stress-responsive gene expression. Solid arrows indicate reported direct or major regulatory relationships, whereas dashed arrows indicate indirect regulation or conceptual connections. This figure was drawn by the authors based on a synthesis of relevant literature and was not directly reproduced or adapted from any single published source.

Among different epigenetic layers and modification types, DNA methylation and histone methylation are more often associated with relatively stable transcriptional regulatory states. By contrast, acetylation is rapid, reversible, metabolism-sensitive, and broad in substrate range. Histone acetylation can regulate chromatin accessibility and transcriptional competence, whereas non-histone acetylation can affect the functional states of transcription factors, metabolic enzymes, signaling proteins, and organellar proteins (Jiang et al., 2020; Kumar et al., 2021; Jin et al., 2024; Wang et al., 2024). Acetylation dynamics are mainly regulated by histone acetyltransferases (HATs) and histone deacetylases (HDACs), and are closely linked to metabolic factors such as acetyl-CoA. Under stress conditions, the redistribution of glycolysis, the tricarboxylic acid cycle, acetate metabolism, and mitochondrial respiration may alter acetyl-CoA production, consumption, and compartmental distribution. Because acetyl-CoA is the direct acetyl-group donor for HAT-mediated acetylation, changes in its availability may further influence both histone and non-histone acetylation. Thus, stress-induced metabolic reprogramming can connect short-term metabolic fluctuations with changes in chromatin accessibility, protein functional states, and stress-response outputs by reshaping acetyl-CoA supply between the cytosol and nucleus (Chen et al., 2017; Lu et al., 2023).

In this context, early Ca²^+^/ROS/ABA signaling may be linked to acetylation regulation through at least two mechanisms. First, hormone- or stress-responsive transcription factors can act as entry points between early signaling and chromatin regulation. For example, in apple, the ABA-responsive transcription factor MdABI5 forms a complex with the histone deacetylase MdHDA6 and promotes histone deacetylation near MdABI5-regulated drought-responsive genes, thereby affecting their expression and drought tolerance (Li et al., 2023). This example suggests that hormone-responsive transcription factors not only transmit upstream signals, but may also provide targeting routes for HAT/HDAC recruitment or functional regulation at specific stress-responsive loci. Second, stress-induced metabolic changes can alter acetyl-CoA availability and thereby influence HAT-mediated acetylation outputs. Together, these examples suggest that early Ca²^+^/ROS/ABA signals may be connected to acetylation regulation through transcription factor-mediated chromatin targeting and stress-induced changes in acetyl-CoA availability. These rapid, reversible, and metabolism-sensitive features make acetylation a suitable focus for linking transient stress signals, metabolic changes, and adaptive regulatory outputs.

Despite increasing evidence that acetylation participates in plant stress responses, its role in stress memory and cross-tolerance has not yet been fully integrated into a unified conceptual framework (Jiang et al., 2020; Kumar et al., 2021; Jin et al., 2024; Wang et al., 2024). It should also be noted that the strength of evidence is uneven across different acetylation-related mechanisms in plants. Evidence for histone acetylation in stress-responsive gene activation, chromatin accessibility, and HAT/HDAC-mediated transcriptional regulation is relatively strong. By contrast, non-histone acetylation in plant stress adaptation is still an emerging area, and some mechanistic concepts are still partly informed by mammalian or yeast systems. Current plant-specific evidence is strongest for defined regulatory modules and substrates, including interactions between stress-responsive transcription factors and HAT/HDAC complexes, acetylation/deacetylation of selected transcription factors, and cytosolic or organellar protein acetylation linked to ion homeostasis, translational regulation, and energy metabolism. Existing studies have often focused separately on histone acetylation, non-histone protein acetylation, stress-responsive transcription, or crop stress tolerance. Therefore, a broader synthesis is needed to clarify how acetylation connects transcriptional memory at the chromatin level, non-histone signaling regulation, metabolic reprogramming, and subcellular stress coordination, and how these mechanisms may be translated into crop resilience improvement (Liu et al., 2022; Lu et al., 2023).

In this review, we propose that acetylation functions as a dynamic regulatory interface linking plant stress memory, cross-tolerance, and crop resilience design. We discuss this interface through four interconnected layers: transcriptional bookmarking at the chromatin level, non-histone signaling regulation, metabolic-epigenetic coupling, and coordination among different subcellular compartments. We then summarize key HAT/HDAC regulatory nodes and their conservation and divergence from model plants to crops, before evaluating the potential and current limitations of acetylation-based regulatory strategies, including priority target selection, small-molecule intervention, synthetic biology, epigenome editing, and molecular design breeding.

## 2 Acetylation as a dynamic regulatory interface for stress memory and cross-tolerance

Acetylation may contribute to plant stress memory and cross-tolerance through several mechanistically distinct but interconnected regulatory layers. At the chromatin level, histone acetylation can increase the transcriptional competence of stress-responsive loci and facilitate rapid gene reactivation (Hu et al., 2019; Wang et al., 2024). At the non-histone level, acetylation and deacetylation can regulate the activity, stability, interaction properties, and subcellular behavior of transcription factors, signaling proteins, metabolic enzymes, and ribosome-associated proteins (Jin et al., 2024). At the metabolic level, acetyl-CoA-dependent and NAD^+^-dependent regulatory processes provide a direct interface between cellular metabolic status and acetylation dynamics (Hu et al., 2019; Lu et al., 2023). At the subcellular level, acetylation of proteins in chloroplasts, mitochondria, and the cytoplasm contributes to photosynthetic maintenance, energy balance, ion homeostasis, translational control, and proteostasis (Xia et al., 2022; Jin et al., 2024). Together, these layers allow acetylation to function not as a single linear mechanism of stress memory, but as a dynamic regulatory interface that links transient stress perception with reprogrammable adaptive states.

### 2.1 Chromatin-level transcriptional bookmarking

At the chromatin level, histone acetylation provides a rapid and reversible mechanism for adjusting the transcriptional competence of stress-responsive loci. By neutralizing the positive charge of lysine residues, acetylation weakens histone-DNA interactions, increases chromatin accessibility, and facilitates the recruitment or retention of transcription factors, RNA polymerase II, and associated transcriptional complexes (Hu et al., 2019; Wang et al., 2024). Histone acetylation marks such as H3K9ac and H3K14ac are therefore closely associated with the activation of stress-responsive genes and may contribute to transcriptional memory by maintaining or re-establishing a chromatin state that is more permissive for rapid gene reactivation (Hu et al., 2019; Kappel et al., 2023).

This chromatin-based mechanism is particularly relevant to stress-responsive promoters that need to be activated quickly upon recurrent stress. After an initial stress exposure, selected loci may retain a more accessible chromatin configuration, enriched histone acetylation, or a transcriptionally poised state. Such a state does not necessarily mean that acetylation marks are permanently maintained as fixed “memory marks.” Rather, current evidence supports a more cautious interpretation: histone acetylation contributes to a permissive chromatin environment that facilitates sustained induction or enhanced re-induction of stress-responsive genes upon subsequent challenge (Kappel et al., 2023; Wang et al., 2024). However, the exact contribution of histone acetylation to transcriptional memory remains difficult to separate from its role in immediate stress activation. Direct evidence showing that blocking a partial acetylation-retention phase, for example through HDAC overexpression, specific mutants, or pharmacological intervention, impairs transcriptional memory is still limited. This distinction between immediate activation and transcriptional memory is important for interpreting the dynamic model proposed below.

**Figure 2:**
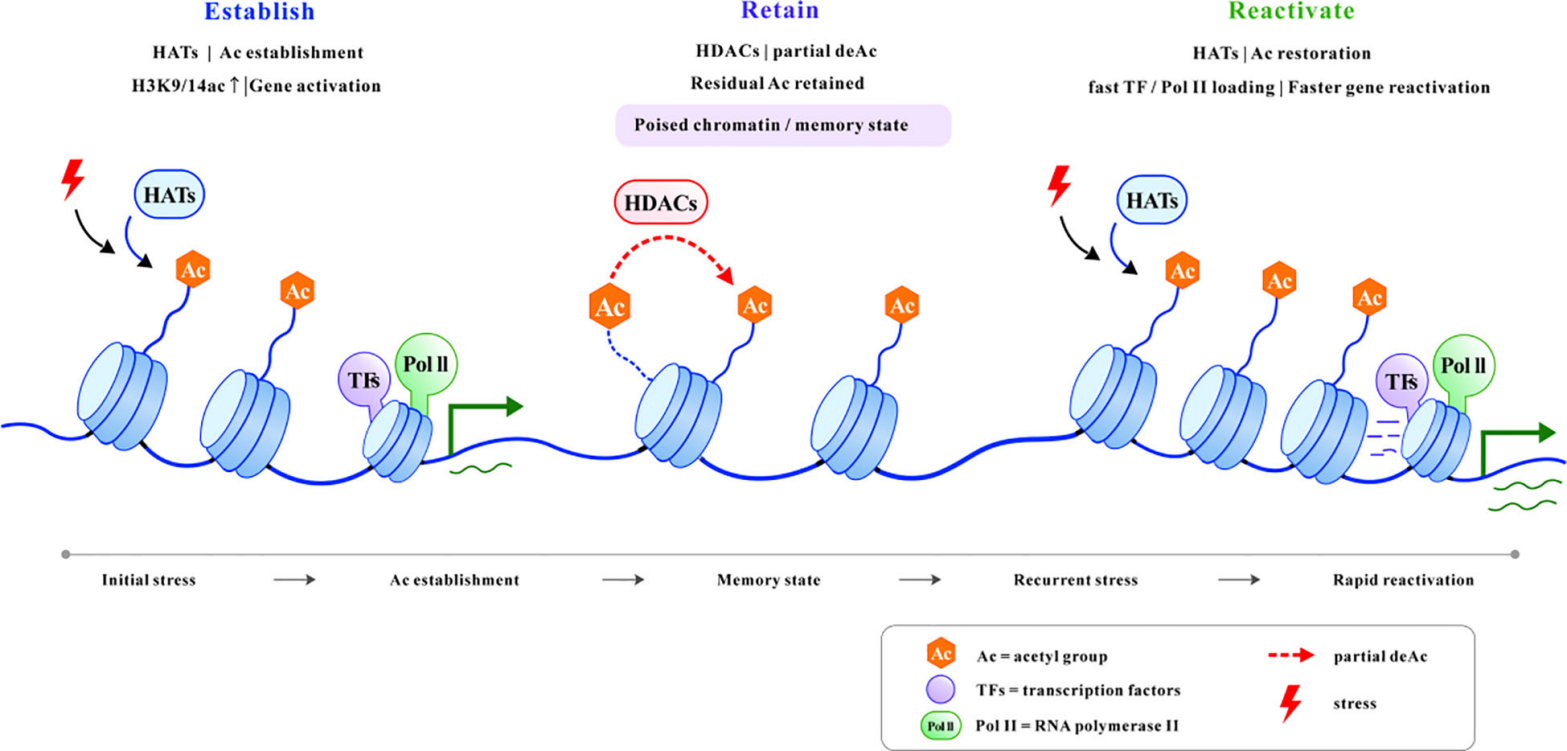
Histone acetylation primes chromatin-based somatic stress memory in plants.

Therefore, the dynamic model proposed below mainly describes chromatin-based stress memory at the somatic level, namely the retention and remobilization of chromatin states induced by stress within the same generation, rather than the stable transmission of parental stress information to offspring. As illustrated in **Figure 2**, we conceptualize this process as three interconnected phases. During the initial stress phase, HAT-mediated acetylation is established at stress-responsive loci, leading to increased H3K9/14ac, enhanced chromatin accessibility, and gene activation. After stress release, HDAC-mediated partial deacetylation may remove part of the acetylation signal, whereas selected loci may retain residual acetylation or relatively high chromatin accessibility, thereby forming a transcriptionally poised state. During recurrent stress, HAT-mediated acetylation restoration may facilitate rapid loading of transcription factors and RNA polymerase II, accelerating the reactivation of stress-responsive genes. This model therefore presents histone acetylation as a dynamic contributor to transcriptional accessibility, poised chromatin states, and rapid reactivation, rather than as a single, permanent molecular carrier of stress-memory information.

A representative example is the drought-responsive regulatory module in Populus trichocarpa. Under drought stress, H3K9ac is increased at the promoters of responsive genes such as *PtrNAC006*, *PtrNAC007*, and *PtrNAC120*, accompanied by RNA polymerase II enrichment and transcriptional activation. This process is mediated by recruitment of the ADA2b-GCN5 complex by the transcription factor PtrAREB1-2, providing a clear example of how a transcription factor-coactivator-HAT module can couple environmental stress perception with promoter-level histone acetylation and gene activation (Li et al., 2019). However, this example mainly supports H3K9ac establishment and transcriptional activation during the initial drought response. Whether this acetylation state is retained after stress release or restored more rapidly during recurrent stress still requires repeated-stress treatments and time-series ChIP analyses.

A similar pattern has been observed in heat-induced transcriptional memory in *Arabidopsis thaliana*. Heat memory genes such as *APX2*, *HSP22*, and *HSA32* can remain transcriptionally poised and retain permissive chromatin features during recovery, enabling faster reactivation upon recurrent heat stress (Friedrich et al., 2021; Kappel et al., 2023; Pratx et al., 2023). This memory state depends on coordinated chromatin and transcriptional features rather than on a single acetylation mark alone.

Thus, histone acetylation is better understood as one of the major foundations at the chromatin level for plant stress memory, rather than as the sole carrier of memory information. Its importance lies in its ability to make stress-responsive loci more transcriptionally competent, thereby helping plants shift from a purely transient response toward a state that can be more rapidly reactivated during recurrent stress (Hu et al., 2019; Wang et al., 2024).

### 2.2 Non-histone acetylation as an emerging signaling switch

The regulatory scope of acetylation extends beyond histones to a broad range of non-histone substrates. Among these, transcription factors represent particularly important functional targets because they directly control downstream stress-responsive gene networks. Acetylation or deacetylation of transcription factors can affect their protein stability, DNA binding capacity, transcriptional activation activity, protein-protein interactions, and subcellular localization (Jin et al., 2024). In this way, non-histone acetylation provides a mechanism through which stress signals can be translated into changes in transcriptional output without relying solely on chromatin remodeling.

From a mechanistic perspective, one important feature of transcription factor acetylation is its potential interaction with ubiquitination. Because both acetylation and ubiquitination occur on lysine residues, these two modifications may compete for the same or nearby sites under certain conditions, thereby influencing protein turnover. Acetylation may also alter protein conformation or interaction states, which can further affect DNA binding and transcriptional activity (Jin et al., 2024). However, these effects are highly protein-specific and context-dependent. Therefore, non-histone acetylation should be regarded as an emerging regulatory layer in plant stress signaling rather than as a universal mechanism applicable to all stress-responsive transcription factors.

**Figure 3:**
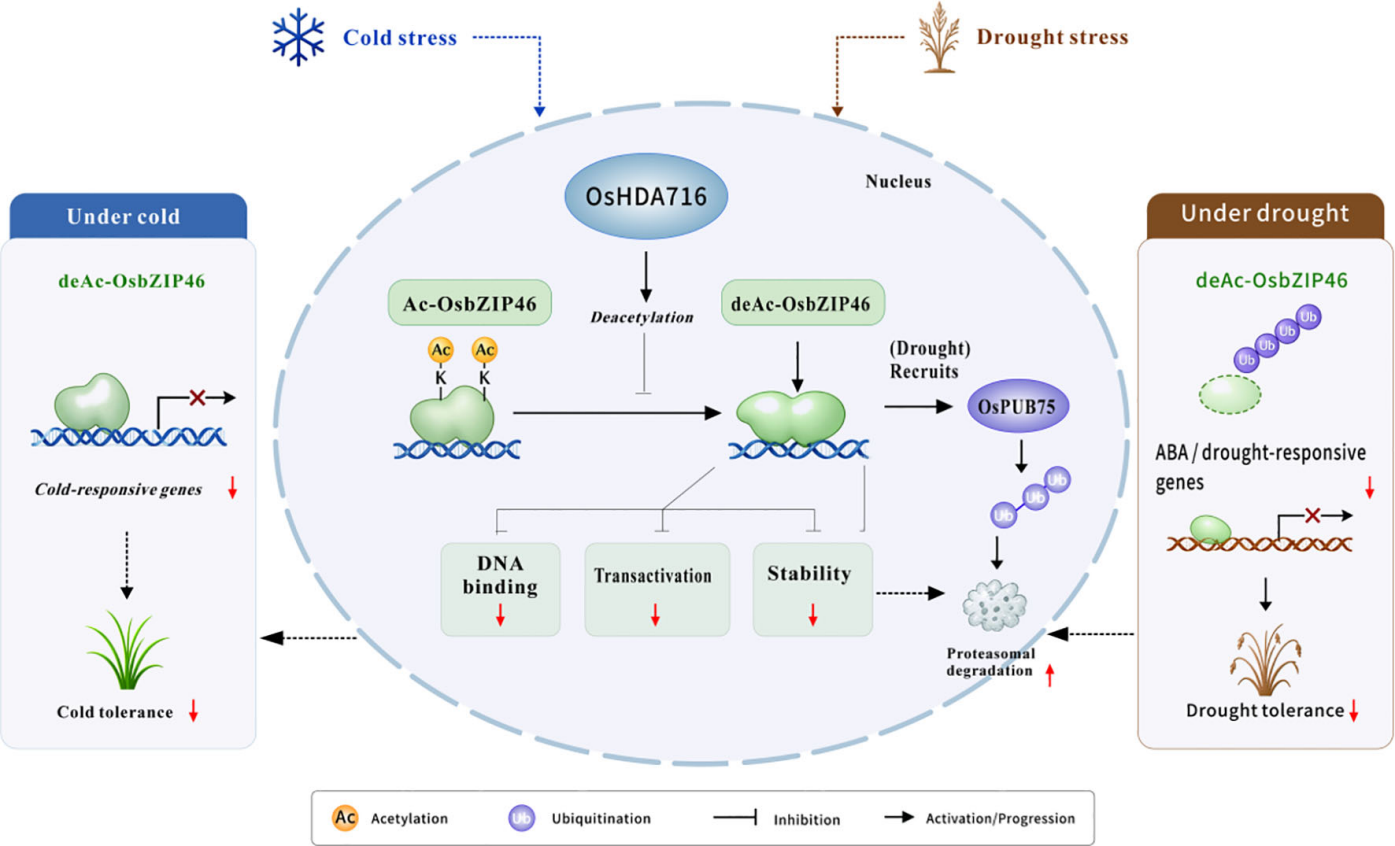
OsHDA716-mediated OsbZIP46 deacetylation suppresses rice cold and drought tolerance.

A well-supported example is the OsHDA716-OsbZIP46 module in rice. OsHDA716 interacts with OsbZIP46 and promotes its deacetylation, thereby reducing the DNA binding capacity, transcriptional activation activity, and protein stability of OsbZIP46. Under cold stress, this regulation weakens the activation of downstream cold-tolerance genes and compromises chilling tolerance (Sun et al., 2024). Under drought stress, OsHDA716 can also recruit the E3 ubiquitin ligase OsPUB75, which cooperatively promotes the deacetylation, ubiquitination, and degradation of OsbZIP46. This suppresses the expression of ABA-responsive and drought-responsive genes and reduces drought tolerance (Sun et al., 2025). Thus, the OsHDA716-OsbZIP46 module provides a representative example of how non-histone deacetylation can regulate both transcription factor activity and protein stability under different stress contexts, thereby connecting acetylation dynamics with stress signaling output (**Figure 3**).

Although the OsHDA716-OsbZIP46 module mainly reveals a molecular mechanism for rice cold and drought responses, whether similar regulatory nodes contribute to natural variation in cold tolerance remains an open question. Natural variation in cold tolerance during germination has been mapped to specific genomic regions in wild rice (Pan et al., 2023). This raises the possibility that allelic variation in acetylation-related regulators, stress-responsive transcription factors, or their downstream targets may contribute to phenotypic diversity in cold tolerance at the germination stage. Thus, the OsHDA716-OsbZIP46 module not only provides a mechanistic example of non-histone acetylation in rice stress responses, but also points to a potential direction for identifying cold-tolerance-related regulatory nodes from natural variation and genetic mapping.

A similar regulatory logic occurs in the Arabidopsis HDA9-*WRKY53* module. In Arabidopsis thaliana, HDA9 interacts with *WRKY53* and promotes its deacetylation, thereby reducing its DNA binding capacity and transcriptional activation activity. This regulation weakens the activation of salt- and drought-responsive genes and increases plant sensitivity to salt and drought stress (Zheng et al., 2020). This example indicates that non-histone acetylation or deacetylation can modulate plant stress responses by altering transcription factor activity. In these cases, acetylated OsbZIP46 or *WRKY53* represents the functionally more active state, whereas HDAC-mediated deacetylation attenuates DNA binding, transcriptional activation, or protein stability.

However, the conservation of this regulatory pattern should still be interpreted with caution. The OsHDA716-OsbZIP46 module provides clear evidence for an HDAC-bZIP regulatory module in rice, whereas the HDA9-*WRKY53* module suggests that similar acetylation/deacetylation-based regulation may also target other stress-related transcription factor families. Nevertheless, whether such HDAC-transcription factor modules are widespread in *Arabidopsis*, tomato, wheat, or other crops remains to be systematically tested. More broadly, HDAC-transcription factor interactions may represent a reusable regulatory logic in plant stress responses, but the interacting partners, target gene networks, and functional outcomes may vary depending on species, stress type, and transcription factor family.

The broader significance of such regulation lies in its potential contribution to cross-tolerance. If the same transcription factor or signaling node is repeatedly regulated by acetylation or deacetylation under different stress conditions, it may serve as a convergence point linking distinct stress pathways. In this sense, non-histone acetylation/deacetylation dynamics may help explain how shared regulatory nodes participate in cross-tolerance. Nevertheless, direct evidence showing persistent acetylation states of transcription factors after stress release remains limited. Therefore, non-histone acetylation/deacetylation dynamics are better understood as a rapid regulatory mechanism that adjusts transcription factor function and signaling output, rather than as fully established stable memory marks. Future studies combining repeated-stress treatments, acetylation-site mutagenesis, protein-stability analysis, and time-resolved acetylome profiling will be needed to test whether such regulatory events contribute directly to stress memory or cross-tolerance (Jin et al., 2024; Sun et al., 2024, 2025).

### 2.3 Metabolic coupling: acetyl-CoA and NAD^+^-dependent deacetylation link metabolic state with stress regulation

Acetylation is closely connected with cellular metabolism because acetyl-CoA serves as the direct donor of acetyl groups for acetylation reactions. Therefore, changes in carbon metabolism, energy metabolism, and acetate-related pathways under stress conditions can influence the level and distribution of histone and non-histone acetylation. This feature distinguishes acetylation from many other regulatory modifications and makes it a potential bridge between metabolic status and stress-responsive gene regulation (Hu et al., 2019; Lu et al., 2023).

Under environmental stress, plants often undergo extensive metabolic reprogramming to maintain energy supply, redox balance, osmotic adjustment, and defense responses. These metabolic changes can alter acetyl-CoA availability, thereby affecting acetylation reactions. In *Arabidopsis*, elevated cytosolic acetyl-CoA promotes histone hyperacetylation, particularly H3K27ac, and is accompanied by changes in transcriptional and metabolic states (Chen et al., 2017). In addition, lysine acetylation of ADA2, a cofactor of the GCN5-containing HAT complex, is sensitive to acetyl-CoA availability and influences GCN5-mediated histone acetylation homeostasis (Yu et al., 2024). These findings indicate that acetyltransferase-associated complexes can themselves function as metabolic-responsive regulatory modules, linking acetyl-CoA supply to transcriptional regulation at the chromatin level.

A more stress-specific example is the acetate-mediated drought tolerance pathway in *Arabidopsis*. In drought-related regulation, HDA6 restricts the expression of the acetate biosynthetic genes *PDC1* and *ALDH2B7* through deacetylation. When HDA6 function is impaired, this repression is released, *PDC1* and *ALDH2B7* expression increases, acetate accumulates, and drought tolerance improves (Kim et al., 2017). Therefore, in this pathway, HDA6 is better understood as a negative regulatory node of acetate biosynthetic genes rather than simply as a positive metabolic switch. The acetate biosynthetic genes *PDC1* and *ALDH2B7* are required for this response, as mutants defective in these genes show increased drought sensitivity. Accumulated acetate may link metabolic changes to chromatin regulation by affecting acetyl-CoA supply and histone H4 acetylation. It can also induce JA and JA-Ile accumulation and prime COI1-mediated JA signaling, making related defense genes more readily inducible during subsequent drought stress. This indicates that acetate does not simply activate JA signaling constitutively, but rather establishes a primed state in which JA-related defense genes can be more rapidly or strongly induced during drought challenge. Therefore, acetate-mediated enhancement of drought tolerance should not be simply attributed to the direct effect of H4 acetylation. A more cautious interpretation is that acetate enhances drought adaptation through two parallel routes: metabolic-epigenetic regulation and JA-related hormonal priming, although current evidence is still insufficient to quantitatively distinguish their relative contributions. This pathway indicates that stress-induced metabolic reprogramming can affect plant drought tolerance through metabolic-hormonal-epigenetic coupling (Kim et al., 2017).

Metabolic control of acetylation is not limited to acetyl-CoA-dependent writing reactions. NAD^+^-dependent sirtuin-type deacetylases also provide a direct link between cellular energy status and stress regulation. In Arabidopsis, AtSRT1 has been shown to coordinate glycolysis, mitochondrial respiration, and stress responses. Mechanistically, AtSRT1 interacts with AtMBP-1, a transcriptional repressor derived from the glycolytic enolase gene *LOS2*/*ENO2*, and negatively regulates glycolysis and stress tolerance while promoting mitochondrial respiration (Liu et al., 2017). This example indicates that deacetylation-based regulation can also be embedded in primary metabolic flux control, thereby linking energy metabolism with stress-responsive transcriptional programs. Because drought, salinity, and heat commonly disturb energy supply, respiratory metabolism, and redox balance, NAD^+^-dependent deacetylases such as AtSRT1 may represent candidate nodes linking metabolic state, energy allocation, and adaptation to multiple stress types. However, their roles in specific cross-tolerance or long-term stress-memory contexts still require further validation.

Taken together, metabolic coupling allows acetylation and deacetylation to function as more than local protein modifications. Through acetyl-CoA-dependent acetylation, acetate-mediated H4 acetylation, JA-related priming, and NAD^+^-dependent sirtuin activity, plants can connect stress-induced metabolic changes with chromatin regulation, hormone signaling, and adaptive physiological responses. This metabolic sensitivity makes acetylation well suited to stress memory and cross-tolerance: it can translate transient metabolic changes caused by stress into regulatory states that remain poised for subsequent activation (Kim et al., 2017; Liu et al., 2017; Hu et al., 2019). However, clear mechanistic evidence for this metabolic-acetylation coupling framework still comes mainly from Arabidopsis and a limited number of representative pathways. Future studies should test the conservation, relative contribution, and practical relevance of these mechanisms across diverse crops, stress types, and combined or recurrent stress conditions.

### 2.4 Compartment-specific acetylation and whole-cell coordination

In addition to its nuclear functions, acetylation is widely distributed across multiple subcellular compartments, including chloroplasts, mitochondria, and the cytoplasm (Finkemeier et al., 2011; Smith-Hammond et al., 2014; Hartl et al., 2017). This spatial distribution is important because plant stress adaptation depends not only on transcriptional regulation, but also on the coordinated maintenance of photosynthesis, respiration, redox balance, ion homeostasis, protein synthesis, and proteostasis. Compartment-specific acetylation therefore provides a cellular basis for integrating local protein regulation with whole-cell stress adaptation (Xia et al., 2022; Jin et al., 2024). From the perspective of stress priming and cross-tolerance, such regulation in different subcellular compartments may provide a functional basis for rapid recovery or primed responses during subsequent stress by shaping metabolic, redox, and protein-synthesis states after stress release.

Chloroplasts are central organelles for photosynthesis and are also major sites of stress perception and redox signaling. Stress-induced changes in chloroplast photosynthesis, ROS production, redox status, and organellar gene expression can be transmitted to the nucleus through chloroplast retrograde signaling, thereby coordinating nuclear stress-response gene expression under environmental stress (Sun and Guo, 2016; Crawford et al., 2018). Chloroplast protein acetylation has been implicated in photosynthetic efficiency, photosystem II (PSII) repair, light-energy distribution, and ROS homeostasis. Under stress conditions, these regulatory effects are closely associated with chloroplast ROS production, redox signaling, and damage-repair processes (Foyer and Hanke, 2022; Li and Kim, 2022). Chloroplast-specific GNAT family acetyltransferases, particularly GNAT2/NSI, participate in light-harvesting complex II (LHCII)-related state transitions and thylakoid membrane dynamics. Disruption of GNAT2/NSI-related regulation can impair LHCII relocation and PSI-LHCII supercomplex formation, leading to imbalanced light-energy distribution and reduced photosynthetic efficiency (Koskela et al., 2018; Rantala et al., 2022). In addition, acetylation can participate in PSII repair through a metabolism-to-translation coupling mechanism. In Chlamydomonas reinhardtii, acetylation of the pyruvate dehydrogenase subunit DLA2 at K197 promotes its functional switch from metabolism to translation regulation, allowing it to bind *psbA* mRNA, promote D1 protein synthesis, and accelerate PSII repair (Neusius et al., 2022). These findings suggest that chloroplast acetylation links metabolic status, photosynthetic protein synthesis, ROS control, and photosystem repair under stress.

Mitochondrial acetylation provides another important layer of stress-related regulation. Mitochondria are major sites of ATP production, respiratory metabolism, and ROS generation, and acetylation of mitochondrial proteins can influence energy metabolism and redox balance. Changes in mitochondrial function can also be communicated to the nucleus through mitochondrial retrograde signaling, which helps coordinate nuclear gene expression with mitochondrial energy and redox status during stress adaptation (Kleine and Leister, 2016; Van Aken et al., 2016). In *Arabidopsis*, *SRT2* localizes predominantly to the inner mitochondrial membrane and interacts with energy metabolism-related complexes, including ATP synthase and ADP/ATP carriers. Loss of *SRT2* changes the acetylation levels of these proteins and alters metabolite abundance, indicating that mitochondrial deacetylation participates directly in the regulation of energy metabolism (König et al., 2014). In cotton, the GhHSP24.7-GhHDA14 module further indicates that mitochondrial protein acetylation can participate in stress-related energy and redox regulation. GhHSP24.7 modulates the mitochondrial deacetylase GhHDA14, thereby altering mitochondrial protein acetylation, ATP content, ROS accumulation, and stomatal conductance under abiotic stress (Ma et al., 2023). These findings indicate that mitochondrial acetylation can influence stress tolerance by modulating ATP production and ROS balance. Under stress-related redox perturbation, ANAC017-mediated mitochondrial retrograde signaling can further help maintain mitochondrial respiratory capacity and cellular redox homeostasis (Fuchs et al., 2022).

At the cytoplasmic level, acetylation regulates stress adaptation through ion transport, signaling components, and translation-associated proteins. In *Arabidopsis*, acetylation of the 14-3–3 protein GRF6 at K56 affects its regulation of the plasma membrane H^+^-ATPase AHA2. Acetylated GRF6 inhibits its interaction with AHA2 and limits pump activation, whereas deacetylation under alkaline stress relieves this inhibition, enhances AHA2 activity, and contributes to ion homeostasis (Guo et al., 2022). This example shows how cytoplasmic acetylation can directly influence membrane transport and stress-related physiological adjustment.

**Figure 4:**
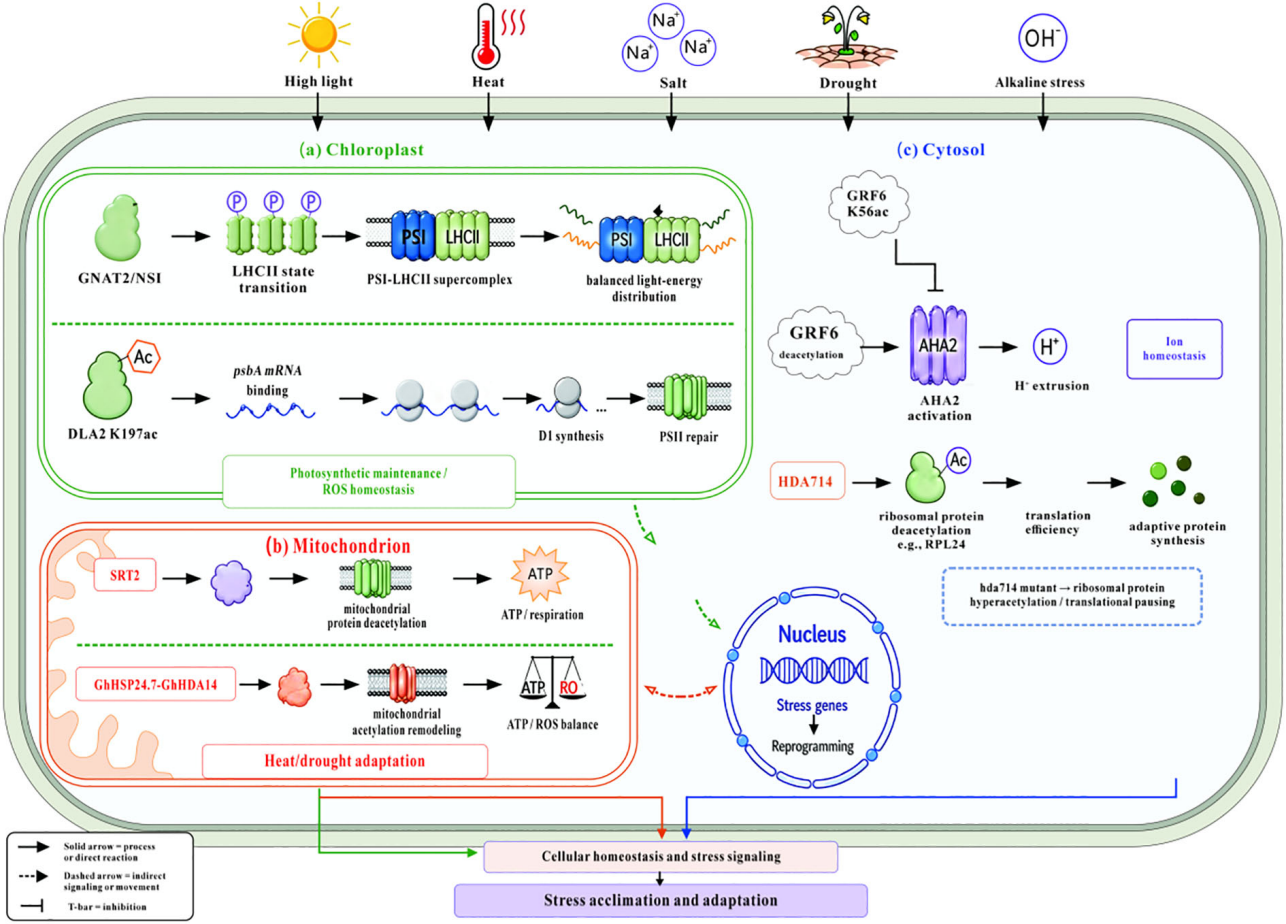
Compartment-specific acetylation coordinates organelle function and whole-cell stress adaptation.

Cytoplasmic deacetylation also affects translational control. In rice, the cytoplasm-localized lysine deacetylase HDA714 modulates the acetylation status of ribosomal proteins. In *hda714* mutants, ribosomal proteins such as RPL24 become hyperacetylated, leading to reduced protein stability, increased translational pausing, and decreased synthesis of stress-responsive proteins (Xu et al., 2021). Under heat stress, HDA714 also contributes to non-histone deacetylation, glycolysis-related metabolic reprogramming, and stress granule formation, thereby helping maintain proteostasis and thermotolerance (Chen et al., 2024). Thus, HDA714 is best interpreted as a cytoplasmic translational-regulatory node rather than a regulator acting at the chromatin level in this context. These regulatory routes in different subcellular compartments are summarized in **Figure 4**.

This figure summarizes how acetylation and deacetylation regulate stress-related processes in chloroplasts, mitochondria, and the cytoplasm. In chloroplasts, GNAT2/NSI-related regulation contributes to LHCII state transitions and balanced light-energy distribution, whereas DLA2 acetylation promotes psbA mRNA binding, D1 synthesis, and PSII repair. In mitochondria, SRT2 and GhHSP24.7-GhHDA14-related regulation affects mitochondrial protein acetylation, ATP production, respiration, and ROS balance. In the cytoplasm, GRF6 deacetylation promotes AHA2 activation and ion homeostasis, while HDA714-mediated ribosomal protein deacetylation contributes to translation efficiency and adaptive protein synthesis. Together, these acetylation processes in different subcellular compartments support cellular homeostasis, stress signaling, and stress acclimation.

Overall, acetylation in different subcellular compartments shows that stress adaptation is not controlled solely by nuclear chromatin regulation. Instead, acetylation coordinates photosynthetic maintenance, mitochondrial energy balance, ROS regulation, ion transport, translational efficiency, and protein homeostasis. Although direct evidence for “organellar acetylation memory” remains limited, organellar and cytoplasmic acetylation may help maintain metabolic, redox, and proteostatic poise after stress release. These recovery-associated states could support faster recovery or primed responses upon subsequent stress and may also feed into chloroplast-to-nucleus and mitochondria-to-nucleus retrograde signaling. In this sense, in different subcellular compartments acetylation provides a mechanistically plausible but still testable layer linking local organelle status with whole-cell stress adaptation (Chen et al., 2017; Hu et al., 2019; Xia et al., 2022; Wang et al., 2024).

## 3 Key regulatory nodes: conservation, functional divergence, and evidence hierarchy

The biological significance of acetylation regulators cannot be judged only by their enzymatic identity as “writers” or “erasers.” Although HATs and HDACs jointly maintain the dynamic balance of acetylation, their functional outputs in plant stress responses are not simply equivalent to “activation” or “repression.” More precisely, the regulatory direction of a given HAT or HDAC depends on the regulatory module in which it operates, its target genes or non-histone substrates, interacting proteins, tissue context, stress type, and species-specific genetic background. Therefore, when discussing acetylation-mediated stress memory and cross-tolerance, the key question is not simply which family members exist, but which members function as evidence-supported regulatory nodes.

### 3.1 Core HAT/HDAC families: from enzymatic identity to regulatory function

Having established acetylation as a regulatory interface for stress memory, cross-tolerance, metabolic coupling, and coordination among different subcellular compartments, we next summarize the enzymatic systems that write and erase acetyl marks. HATs use acetyl-CoA as the acetyl donor to acetylate lysine residues on histones and, in some cases, non-histone substrates. Although these enzymes can also be broadly referred to as lysine acetyltransferases (KATs), we use HATs throughout this review for consistency with most plant epigenetic and stress-response studies. Through these reactions, HATs influence chromatin accessibility, transcriptional-complex recruitment, and protein functional states. Plant HATs are generally classified into GNAT/HAG, MYST/HAM, p300/CBP/HAC, and TAFII250/HAF subfamilies. Among them, GNAT/HAG members, especially GCN5-like proteins have been relatively well studied in stress-induced transcriptional activation, whereas HAC and HAF members are characterized by complex domain architectures and potential transcriptional coactivator functions (Pandey et al., 2002; Jiang et al., 2020; Kumar et al., 2021).

HDACs remove acetyl groups from lysine residues and participate in chromatin-state resetting, transcriptional repression, protein stability control, and metabolic or signaling regulation. Plant HDACs generally include three classes: RPD3/HDA1, HD2/HDT, and SIR2/SRT. Among them, RPD3/HDA1 members are relatively numerous and broadly involved in development, hormone responses, immunity, and abiotic stress responses. HD2/HDT proteins are plant-specific deacetylases and are often associated with nuclear transcriptional regulation, ribosome biogenesis, and coordination between development and stress responses. SIR2/SRT members are NAD^+^-dependent and therefore more directly connect energy metabolic status with deacetylation-based regulatory outputs (Pandey et al., 2002; Luo et al., 2012; Liu et al., 2014; Yruela et al., 2021).

**Figure 5:**
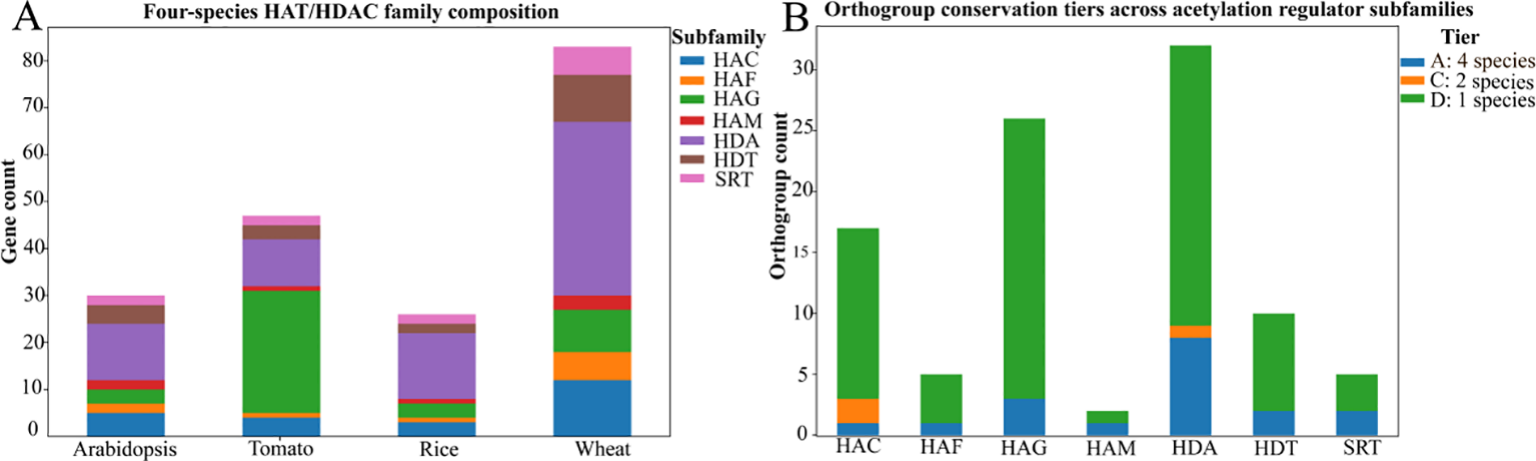
Conservation and divergence of HAT/HDAC regulators in model plants and representative crops. **(A)** Subfamily composition of HAT/HDAC regulators in Arabidopsis thaliana, tomato, rice, and wheat. HAT subfamilies include HAC, HAF, HAG, and HAM, whereas HDAC subfamilies include HDA, HDT, and SRT. **(B)** Orthogroup conservation tiers within HAT/HDAC subfamilies. Orthogroups were classified as four-species conserved, two-species shared, or species-specific. These comparisons indicate that plant acetylation regulators retain a conserved enzymatic framework while showing subfamily- and species-specific expansion. Gene numbers should be interpreted in light of genome size, ploidy level, and annotation criteria.

Comparing HAT/HDAC family composition among different species can provide a background for understanding the conservation and divergence of acetylation regulatory systems. As shown in **Figure 5A**, Arabidopsis, tomato, rice, and wheat all retain the major HAT and HDAC subfamilies, indicating that acetyl-group writing and erasing systems are broadly conserved in plants. However, their copy-number distributions differ markedly among subfamilies and species. For example, the HAG branch is clearly expanded within the tomato HAT family, whereas wheat shows a strong overall increase in both HAT and HDAC members, likely because of its polyploid genome background. **Figure 5B** further shows that orthogroup conservation is uneven across HAT/HDAC subfamilies. Some orthogroups are retained across all four species, whereas others are shared by only two species or retained in a species-specific manner. These comparisons indicate that plant acetylation regulatory systems have both a conserved enzymatic backbone and clear lineage-specific expansion and divergence.

It should be emphasized that family size or orthogroup conservation does not directly equal functional importance. Whether a family member becomes a key node in stress memory or cross-tolerance further depends on whether it is induced under specific stress conditions, whether it targets key chromatin loci or non-histone substrates, whether it is embedded in abscisic acid (ABA), JA, ROS, Ca²^+^, submergence, or other signaling pathways, and whether its regulation can produce stable physiological or agronomic phenotypes. Therefore, HAT/HDAC family analysis can only provide a background for candidate nodes. The real functional interpretation must return to specific regulatory modules and levels of evidence.

### 3.2 Conserved regulatory modules in model plants and crops

Building on the conserved and divergent features of HAT/HDAC families described above, this section further focuses on representative regulatory modules that have been functionally characterized in model plants and crops, and discusses how these modules connect acetylation dynamics, stress signaling, and adaptive outputs (Luo et al., 2017; Yruela et al., 2021). In plant stress responses, acetylation/deacetylation is not controlled by a single enzyme or target site. Instead, it is formed by a regulatory network composed of HATs, HDACs, substrates, interacting proteins, and downstream signaling pathways (Pandey et al., 2002; Luo et al., 2012; Yruela et al., 2021). To avoid simply listing multiple gene examples side by side, a more reasonable approach is to classify currently better-supported nodes into several regulatory modules according to their mechanistic logic. It should be noted that not all key nodes discussed in this section have been directly proven to participate in stress memory or cross-tolerance. Some of them already have relatively clear evidence related to stress response, stress tolerance, or priming, whereas others should be viewed as candidate modules with potential relevance to stress memory or cross-tolerance because they regulate shared signaling pathways, chromatin states, metabolic status, or non-histone protein functions. Therefore, these nodes are better understood according to different levels of evidence, rather than being simply treated as fully validated “memory regulators” or “cross-tolerance factors” (Lämke and Bäurle, 2017; Oberkofler et al., 2021; Kappel et al., 2023).

Stress tolerance, stress memory, and cross-tolerance are not isolated concepts. Previous studies have shown that mild stress pretreatment can enhance plant responses to subsequent challenges. When the later challenge is the same or closely related stress, this response is better described as stress memory or priming-induced enhanced reactivation; when the later challenge is a different stress, the phenomenon is more appropriately described as cross-tolerance. For example, drought pretreatment can maintain higher antioxidant enzyme activity, photosynthesis-related protective components, or metabolic preparedness during the recovery phase, thereby supporting faster responses when plants encounter stress again. Heat, drought, or combined-stress priming can also enhance antioxidant defense, osmotic adjustment, and photosynthetic protection, improving the tolerance of crops such as maize to subsequent stress (Ru et al., 2022). In *Pinus radiata*, maternal drought experience can enhance offspring responses to heat stress, suggesting that parental stress history may contribute to heterologous stress tolerance in the next generation. This observation is more conservatively interpreted as an intergenerational stress effect, whereas stable transgenerational inheritance would require evidence of persistence in later, non-exposed generations. These responses often involve shared nodes such as ROS, Ca^2+^, ABA/JA signaling, antioxidant systems, metabolic reprogramming, and chloroplast functional protection (Perez and Brown, 2014; Nair et al., 2022; Ru et al., 2022; Das and Adak, 2025).

In addition to conventional stress pretreatments, nanoparticle treatment has also emerged as an exogenous strategy for inducing cross-tolerance to multiple stresses in crops. Recent review evidence indicates that nanoparticles can enhance crop adaptation to both biotic and abiotic stresses by modulating shared processes such as ROS homeostasis, antioxidant defense, hormone signaling, nutrient uptake, and metabolic reprogramming (Abdulraheem et al., 2026). However, the above cross-tolerance or externally induced priming examples mainly support the phenomenon of enhanced adaptive capacity after pretreatment. Nanoparticle-induced cross-tolerance should therefore be considered an emerging priming-related phenomenon. Whether it involves acetylation-mediated stress memory remains unresolved and requires direct acetylome, chromatin, and repeated-stress evidence. **A**cetylation/deacetylation regulation deserves attention for two main reasons. First, many different stresses share regulatory nodes such as ROS, Ca²^+^, ABA/JA signaling, antioxidant systems, metabolic reprogramming, and chloroplast functional protection, and HATs, HDACs, and their non-histone substrates can modulate these nodes; therefore, they may influence the connections among different stress-response pathways (Jiang et al., 2020; Zhang et al., 2024). Second, acetylation is rapid, reversible, and may be partially retained. During an initial stress, HAT-mediated acetylation can promote chromatin opening and transcriptional activation at stress-responsive gene loci. After stress release, selected loci may retain residual acetylation, higher chromatin accessibility, or a transcriptionally poised state. Upon recurrent stress, these loci can recruit transcription factors and RNA polymerase II more rapidly, thereby enabling faster gene reactivation (Oberkofler et al., 2021; Kappel et al., 2023).

Consistent with this proposed memory-related role, more direct evidence supporting the involvement of acetylation in stress priming comes from salt-stress priming in soybean. Salt pretreatment significantly alters the genome-wide distribution of H3K9ac, H3K4me2, and H3K4me3 in soybean seedlings and reshapes gene networks related to ion homeostasis, cell-wall modification, and stress responses. More importantly, pharmacological alteration of histone acetylation can induce priming-like transcriptional responses in plants without prior salt pretreatment, suggesting that active chromatin marks such as H3K9ac may participate in the formation of transcriptional states that can be remobilized after pretreatment (Yung et al., 2022). A similar acetylation-related priming logic has also been observed in plant immunity. In Arabidopsis systemic acquired resistance, priming events can establish active chromatin features, including H3/H4 acetylation and H3K4 methylation, at defense-related loci. In next-generation SAR, promoters of genes such as *PR-1*, *WRKY6*, and *WRKY53* can also show H3K9ac enrichment, accompanied by a primed state of SA-dependent defense responses (Jaskiewicz et al., 2011; Luna et al., 2012). These examples provide more direct support than most single-stress tolerance studies for the idea that acetylation participates in primed chromatin states. However, whether this logic is broadly applicable to abiotic stress memory under drought, salinity, and heat still requires more repeated-stress experiments and recovery-phase dynamic analyses.

**Figure 6:**
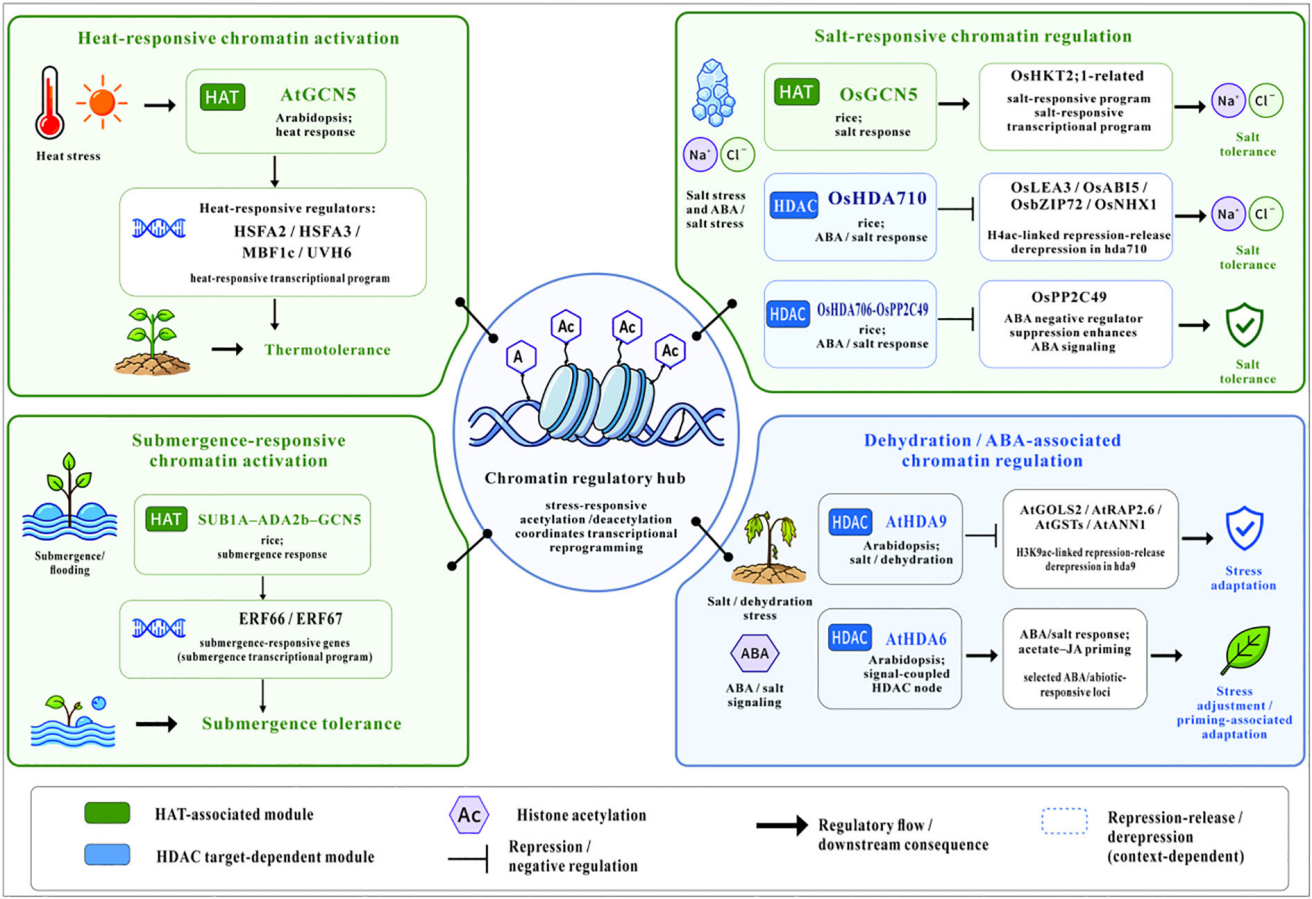
Stress-responsive HAT and HDAC modules coordinate chromatin-linked transcriptional programs.

**Figure 7:**
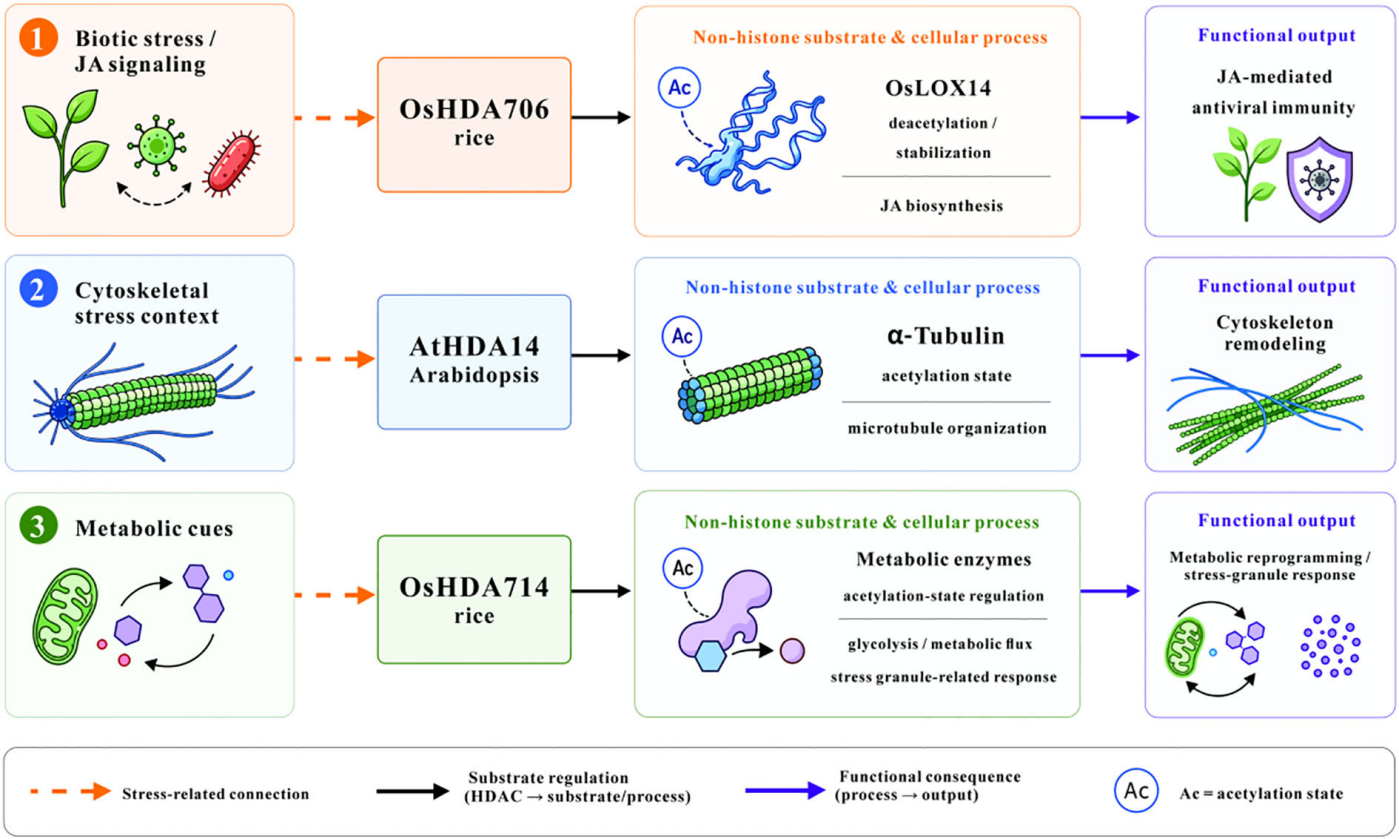
Non-histone and signal-coupled acetylation/deacetylation modules in plant stress responses.

Overall, these regulatory modules can be summarized as two complementary layers of a signal-coupled acetylation/deacetylation network in plant stress responses. As summarized in **Figure 6**, the first layer consists of chromatin-centered HAT/HDAC modules that connect reported stress contexts with histone acetylation or deacetylation at representative stress-responsive loci, thereby influencing transcriptional outputs and adaptive responses (Jiang et al., 2020). As summarized in **Figure 7**, the second layer consists of non-histone and signal-coupled deacetylation modules that regulate protein stability, enzyme activity, cytoskeletal organization, metabolic flux, and stress-granule-related processes (Jiang et al., 2020; Zhang et al., 2024). It should be noted that the modules shown in **Figures 6** and **7** include both regulatory nodes that have been shown to participate in specific stress responses and candidate nodes with potential relevance to stress memory or cross-tolerance; the strength of evidence is not the same across different modules.

The first category is the HAT-mediated transcriptional activation module, represented by GCN5/ADA2b-associated complexes within broader GCN5-containing transcriptional coactivator systems (Wu et al., 2023). The basic logic of this module is that stress signals or specific transcription factors recruit HAT complexes to stress-responsive gene loci, increase local H3/H4 acetylation levels, and thereby enhance transcriptional activation and stress-response outputs. In *Arabidopsis thaliana*, GCN5 is one of the relatively well-studied HATs. Loss of GCN5 weakens the activation of heat-responsive genes and reduces thermotolerance, indicating that GCN5-mediated acetylation activation is an important component of heat stress-induced transcriptional reprogramming (Hu et al., 2015). In rice, *OsGCN5* catalyzes H3/H4 acetylation. Loss of *OsGCN5* inhibits growth and weakens salt tolerance, whereas overexpression enhances seedling adaptation to salt stress, suggesting that GCN5-like HATs have a relatively clear positive regulatory role in crop salt-stress transcriptional output (Xue et al., 2024). In addition, during rice submergence responses, SUB1A-1 can recruit the ADA2b–GCN5 complex and promote the epigenetic activation of downstream genes such as *ERF66* and *ERF67*, thereby enhancing submergence tolerance (Lin et al., 2023). This “transcription factor-coactivator-HAT” module is important because it integrates stress-signal recognition, histone acetylation, and transcriptional output into a single functional unit. It is currently one of the best-supported models of HAT-mediated stress responses. For stress memory, the importance of this type of module mainly lies in the possibility that HAT-mediated chromatin opening and transcriptional activation can provide a potential chromatin basis for rapid re-induction of stress-responsive genes under repeated or heterologous stress. However, in most crop examples, whether this module truly mediates long-term stress memory or cross-tolerance still needs to be further tested through repeated-stress treatments, cross-stress combinations, and tracking of acetylation states after stress release (Oberkofler et al., 2021; Kappel et al., 2023).

The second category is the repression-release module, in which certain HDACs constrain stress-responsive genes with positive roles in stress tolerance. This is consistent with the broader view that plant HDACs can act as transcriptional repressors in abiotic stress responses, although their final effects may be positive or negative depending on their targets and stress contexts (Luo et al., 2017; Cui et al., 2023). In this mode, HDAC activity reduces acetylation at stress-responsive loci, thereby limiting the expression of nearby or target genes. When the corresponding HDAC function is weakened or lost, acetylation at these loci increases, the expression of these stress-responsive genes is derepressed, and stress adaptation is ultimately enhanced. For example, *Arabidopsis* HDA9 is considered an important negative regulator of salt and drought stress responses. Loss of *HDA9* leads to the upregulation of multiple stress-responsive genes and enhances stress adaptation (Zheng et al., 2016). In rice, *OsHDA710* also participates in ABA-mediated salt stress regulation. Loss of *OsHDA710* increases acetylation levels in multiple salt-responsive gene regions and enhances salt tolerance (Ullah et al., 2021). These examples show that HDACs are not necessarily “stress-tolerance-promoting factors.” In some cases, they function more like “brake nodes” that limit the intensity of stress responses. From the perspective of stress memory, the repression-release module may make stress-responsive genes easier to reactivate under subsequent stress by increasing the accessibility of these loci. However, current evidence mainly supports stress tolerance. Whether this module participates in the maintenance of chromatin states after stress release and in rapid reactivation during repeated stress still needs to be further demonstrated by time-series ChIP-seq, transcriptome profiling, and repeated-stress experiments.

The third category is the negative-regulator suppression module, represented by the OsHDA706–OsPP2C49 pathway. Unlike the repression-release module, the positive stress-tolerance effect of *OsHDA706* is not achieved by derepressing stress-tolerance-promoting genes. Instead, it is achieved by deacetylating and suppressing a negative regulatory node in ABA signaling, thereby enhancing stress-response output. Studies have shown that OsHDA706 enhances rice salt tolerance by regulating the deacetylation of H4K5ac/H4K8ac at *OsPP2C49*-associated loci, indicating that deacetylation can amplify ABA-related stress signals by suppressing a negative regulator (Liu et al., 2023). In addition, under viral infection, OsHDA706 can deacetylate and stabilize the JA biosynthetic enzyme OsLOX14, thereby enhancing broad-spectrum antiviral immunity. This further suggests that the same HDAC node can be embedded into hormone metabolism and immune networks through different substrates (Yang et al., 2024). Therefore, the final phenotypic output of an HDAC depends on its target: if it deacetylates and represses stress-tolerance-promoting genes, it may reduce tolerance; if it targets negative regulatory factors, it may enhance tolerance. Because ABA is a core signal shared by several abiotic stresses such as salt, drought, and cold, the OsHDA706–OsPP2C49 module has potential relevance to cross-tolerance (Perez and Brown, 2014; Nair et al., 2022). The OsHDA706–OsLOX14 pathway further suggests that the same HDAC node can connect abiotic-stress and biotic-stress-related networks. However, these results are still better interpreted as candidate evidence for cross-stress signal integration. Whether this module truly mediates the protective effect of one stress pretreatment against another stress still needs to be tested through salt–drought, salt–cold, or abiotic stress–pathogen stress combination experiments.

In addition to the three modules above, HDA6 is better regarded as a signal-coupled HDAC node. In *Arabidopsis*, HDA6 participates in ABA and salt stress responses and affects the histone acetylation status of ABA/abiotic stress-responsive loci, indicating that deacetylation-based regulation can be directly embedded in hormone-related transcriptional programs (Chen et al., 2010; Vincent et al., 2022). At the same time, HDA6 is also associated with acetate metabolism, JA priming, and drought adaptation, suggesting that its role is not limited to a single stress or a single pathway (Kim et al., 2017; Vincent et al., 2022). Rather than simply classifying HDA6 as a positive or negative regulator, it is better interpreted as a multi-signal-coupled node connecting ABA, JA, metabolic status, and chromatin regulation. Compared with single stress-tolerance nodes, HDA6 is a stronger candidate for memory-related regulation because it links acetate metabolism, JA priming, ABA/salt responses, and chromatin acetylation. These features may allow HDA6 to convert metabolic and hormonal signals into a primed state, although its roles in long-term stress memory, cross-stress protection, and transgenerational effects still need to be clarified. These chromatin-centered HAT/HDAC modules are summarized in **Figure 6**, which highlights their reported stress contexts, representative substrates or response programs, and adaptive outputs.

This panel summarizes representative HAT/HDAC modules acting at the chromatin level that link reported stress contexts to specific substrates or response programs and adaptive outputs. AtGCN5 represents an Arabidopsis heat-response HAT module associated with heat-responsive regulators such as HSFA2, HSFA3, MBF1c, and UVH6. OsGCN5 represents a rice salt-response HAT module associated with H3/H4 acetylation and salt-responsive transcriptional regulation, including an OsHKT2;1-related salt-response program. The SUB1A-ADA2b-GCN5 module represents a rice submergence-response pathway that promotes the epigenetic activation of *ERF66* and *ERF67*. The HDAC-related modules illustrate target-dependent regulatory logic: AtHDA9 and OsHDA710 represent repression-release modules acting on stress-responsive genes, AtHDA6 represents a signal-coupled HDAC node associated with ABA/salt responses and acetate–JA priming, and OsHDA706-OsPP2C49 represents suppression of an ABA negative regulator. Stress terms indicate reported experimental or signaling contexts rather than universal upstream inputs. HAT/HDAC outputs are target- and context-dependent, and the modules shown differ in evidence strength.

Notably, acetylation regulatory modules are not limited to transcriptional output at the chromatin level. They can also regulate cell structure, hormone metabolism, and protein homeostasis through non-histone substrates, as summarized in **Figure 7**. For example, HDA14 can influence microtubule stability and cellular architecture through α-tubulin deacetylation (Tran et al., 2012). OsHDA706 can participate in JA biosynthesis and antiviral immunity through the deacetylation and stabilization of OsLOX14 (Yang et al., 2024). OsHDA714 can affect glycolysis-related metabolic reprogramming and stress granule formation through non-histone deacetylation (Chen et al., 2024). These processes indicate that HAT/HDAC nodes control not only nuclear gene expression but also stress adaptation through non-histone routes. For stress memory and cross-tolerance, these non-histone regulatory processes may not directly serve as “memory marks.” However, by maintaining the cytoskeleton, metabolic flux, protein homeostasis, and hormone metabolism, they may provide a basis for rapid physiological adjustment when plants encounter stress again. Therefore, they can be viewed as a functional layer supporting systemic adaptation and cross-stress mobilization.

This panel summarizes representative HDAC-centered modules that regulate non-histone substrates or cellular processes. OsHDA706 can deacetylate and stabilize OsLOX14, thereby supporting JA biosynthesis and antiviral immunity. AtHDA14 is associated with α-tubulin acetylation status and cytoskeletal remodeling. OsHDA714 represents a metabolic regulation-related node linked to metabolic enzyme acetylation, glycolytic flux, and stress-granule-related responses. Dashed orange arrows indicate stress-related connections. Black arrows indicate substrate regulation, and purple arrows indicate functional consequences. Ac indicates acetylation state. The displayed modules differ in evidence strength and stress specificity and should not be interpreted as equivalent or universally conserved pathways.

Taken together, the key to understanding acetylation regulatory networks is not whether a given enzyme is a HAT or an HDAC, but which regulatory logic it enters. HAT-mediated activation modules mainly amplify positive transcriptional output. Repression-release modules enhance responses by weakening HDAC-mediated repression of positive stress-response genes. Negative-regulator suppression modules indirectly increase stress output by deacetylating negative regulatory nodes. Signal-coupled nodes and non-histone functional remodeling modules further bring hormone signaling, metabolic status, protein stability, and cell-structure regulation into the same network. This modular interpretation helps explain why the same class of deacetylases can produce opposite phenotypic outcomes under different stresses, with different targets, and in different species. More importantly, this classification also provides an evidence-level framework for connecting “stress-response nodes” with “stress memory/cross-tolerance nodes.” Not all nodes have been directly proven to participate in stress memory or cross-tolerance, but through shared signaling, chromatin accessibility, metabolic priming, and non-histone functional remodeling, they constitute the major candidate entry points through which acetylation regulation may participate in complex stress adaptation.

It should be noted that the categories in the following table are not strictly mutually exclusive systematic classifications. Rather, they are summarized according to the dominant regulatory logic of acetylation regulators reported in existing studies. The same HAT/HDAC node may fall into different categories depending on its target, substrate type, or stress context. The “association level” does not mean that all nodes have been directly proven to participate in stress memory or cross-tolerance. Instead, it summarizes their relationship with chromatin reactivation, shared stress signaling, metabolic priming, or systemic adaptation.

To clarify the evidence hierarchy of these acetylation-related nodes, **Table 1** summarizes the representative examples according to their dominant regulatory logic rather than by HAT/HDAC family classification. This framework helps distinguish nodes with relatively direct stress-tolerance evidence from those that remain candidate links to stress memory or cross-tolerance.

**Table 1:** Regulatory logic and evidence hierarchy of acetylation-related nodes in plant stress adaptation.

Regulatory logic	Representative nodes	Evidence level	Key limitation	Representative references
HAT-mediated activation	GCN5, ADA2b–GCN5, OsGCN5, SUB1A–ADA2b–GCN5	Mechanistically relevant; mostly candidate-level	Repeated- stress and cross-stress evidence remains limited	(Hu et al., 2015; Lin et al., 2023; Wu et al., 2023; Xue et al., 2024)
HDAC-mediated release of repression	AtHDA9, OsHDA710	Mainly supports stress tolerance	Memory- related chromatin retention remains unclear	(Zheng et al., 2016; Luo et al., 2017; Ullah et al., 2021)
HDAC suppression of negative regulators	OsHDA706– OsPP2C49	Potentially relevant to cross-tolerance	Cross-stress protection still needs direct testing	(Liu et al., 2023; Yang et al., 2024)
Signal-coupled HDAC node	AtHDA6	Candidate memory-related node	Short-term priming and long-term memory need to be distinguished	(Chen et al., 2010; Kim et al., 2017; Vincent et al., 2022)
Non-histone functional remodeling	AtHDA14, OsHDA706–OsLOX14, OsHDA714	Indirect support for systemic adaptation	Causal links from acetylation sites to phenotypes remain incomplete	(Tran et al., 2012; Yang et al., 2024) (Chen et al., 2024; Zhang et al., 2024)

This table summarizes acetylation-related nodes according to their dominant regulatory logic and current evidence level, rather than by HAT/HDAC family classification. The representative references listed in the last column indicate primary studies or directly relevant reviews supporting the corresponding nodes or regulatory logic. “Candidate-level” indicates that a node has mechanistically relevant evidence, such as genetic perturbation, altered acetylation status, target-gene expression changes, or stress-tolerance phenotypes, but has not yet been directly shown to mediate regulatory-state retention after stress release, rapid reactivation during recurrent stress, or heterologous stress protection. A node could be elevated from “candidate-level” to “validated-level” if repeated-stress or cross-stress experiments demonstrate a clear stress-memory or cross-tolerance phenotype and if time-resolved acetylation analysis, genetic complementation, target-site mutation, or causal acetylation-site validation confirms its functional role. If the effect persists in non-exposed progeny across subsequent generations, it would provide stronger evidence for transgenerational stress memory.

### 3.3 Crop-specific divergence and translational boundaries

From the perspective of conservation, HATs and HDACs, as the core enzymatic systems maintaining the dynamic balance of acetylation, show a relatively consistent basic organizational logic in *Arabidopsis*, rice, wheat, tomato, and other plants. GCN5-like HATs, RPD3/HDA1-type HDACs, and some regulatory nodes associated with ABA, JA, heat stress, salt stress, and pathogen responses can all be embedded in hormone signaling, transcriptional reprogramming, and protein functional regulation across different species (Jiang et al., 2020; Wang et al., 2024). However, what is conserved is the regulatory logic; this does not mean that functional strength or phenotypic output is fully conserved. In other words, the “enzymatic backbone” of acetylation regulation can be retained across species, but whether a given node ultimately affects salt tolerance, drought adaptation, submergence response, fruit ripening, or disease resistance often depends on the specific crop background.

This difference is especially clear between model plants and crops. Studies in *Arabidopsis* have mainly revealed the basic logic by which HATs and HDACs participate in stress-responsive transcriptional regulation, hormone-signal coupling, and chromatin remodeling. Rice studies, in contrast, highlight more clearly the functional outputs of these nodes in salt, drought, heat, submergence, and pathogen responses. For example, OsGCN5, OsHDA706, OsHDA710, and the SUB1A–ADA2b–GCN5 module illustrate acetylation-related regulation in cereal stress adaptation from the perspectives of transcriptional activation, negative-regulator suppression, repression release, and submergence response, respectively (Ullah et al., 2021; Lin et al., 2023; Liu et al., 2023; Xue et al., 2024). By contrast, acetylation/deacetylation networks in tomato are more clearly intertwined with developmental transition, fruit ripening, quality formation, and the coordination of disease/stress resistance.

In tomato, SlHDA1 can positively regulate drought and salt tolerance through ABA-related pathways, while also acting as a negative regulator of fruit ripening by affecting ethylene biosynthesis and carotenoid accumulation (Guo et al., 2017b; Guo and Wang, 2023). SlHDA3 and SlHDA7 are also associated with fruit ripening rate, shelf life, and H4ac deacetylation (Guo et al., 2018; Zhou et al., 2024). Among HD2-type deacetylases, *SlHDT1* generally acts as a negative regulator of fruit ripening, and suppression of *SlHDT1* can increase fruit yield but decrease drought and salt tolerance (Guo, 2022; Guo and Wang, 2023). *SlHDT3*, however, shows a different regulatory direction in ripening control, as silencing of *SlHDT3* delays fruit ripening and suppresses carotenoid accumulation (Guo et al., 2017a). In addition, the GCN5-related HAT SlHLS2 participates in tomato fungal resistance and growth regulation, suggesting that HAT family members in horticultural crops may coordinate both development and disease resistance (Jaiswal et al., 2025). Therefore, in crops such as tomato, acetylation-related nodes cannot be evaluated only by whether they enhance stress tolerance. Their effects on ripening, quality, shelf life, yield, and disease resistance must also be considered.

This crop specificity also defines the boundary for translating conclusions from model plants into crop applications. First, regulatory relationships established in model plants cannot be directly equated with functional direction in crops. Even when a HAT/HDAC homolog is relatively conserved at the sequence level, its expression pattern, interacting proteins, target genes, substrate preference, and tissue specificity may change. Second, even if the regulatory direction is broadly conserved, its effect on final agronomic traits may still shift because of differences in growth cycle, organ use, and breeding objectives. For example, priority traits in cereal crops may include root water uptake, ion homeostasis, photosynthetic recovery, and yield stability, whereas horticultural crops such as tomato must balance ripening, quality, and disease/stress resistance.

Therefore, a key regulatory node is not equivalent to a target that can be directly used in breeding. For an acetylation regulator to truly enter crop stress-resilience design, several conditions need to be met: its mechanism and targets should be relatively clear; its stress-related phenotypic direction should be stable; its effect should be reproducible across different genetic backgrounds; its effect should remain robust under multiple environments; and its negative effects on growth, development, yield, and quality should be controllable. In other words, model plant studies provide candidate families, candidate modules, and possible directions of action, rather than directly transferable application conclusions. Only after mechanistic revalidation, phenotypic reassessment, and multi-environment stability testing in the target crop can an acetylation-related node move from being a “key regulatory factor” to a truly breeding-relevant regulatory target.

Overall, plant acetylation regulatory networks show the coexistence of a conserved backbone and crop-specific functional reshaping. Conservation provides a basis for cross-species comparison and candidate-node screening, whereas functional divergence reminds us that the real application potential of these nodes must be evaluated within specific crops, organs, and stress contexts. Based on this understanding, future crop stress-resilience improvement should not simply aim to “increase acetylation” or “inhibit deacetylation.” Instead, it should focus on precise intervention in modules with clear regulatory logic, complete evidence chains, and strong alignment with target crop traits.

## 4 Crop stress-resilience improvement: from acetylation regulatory modules to application boundaries

Acetylation regulatory networks provide new candidate targets and intervention ideas for improving crop stress resilience. However, their application value should not be simply understood as “increasing acetylation” or “inhibiting deacetylation.” As discussed above, the functional direction of HATs and HDACs depends on specific regulatory modules, target genes or substrates, stress types, and crop backgrounds. Therefore, when moving from basic mechanisms to crop application, the key question is not whether an enzyme belongs to HATs or HDACs. Instead, it is whether this node has a clear evidence chain, a stable stress-tolerance phenotype, clear crop relevance, and controllable side effects on growth and quality. Based on this understanding, acetylation-related crop improvement strategies can be considered mainly from four aspects: priority target selection, small-molecule regulation, epigenome editing/synthetic module design, and molecular design breeding.

### 4.1 Prioritizing regulatory targets: evidence strength, crop relevance, and stress-resilience improvement

**Figure 8:**
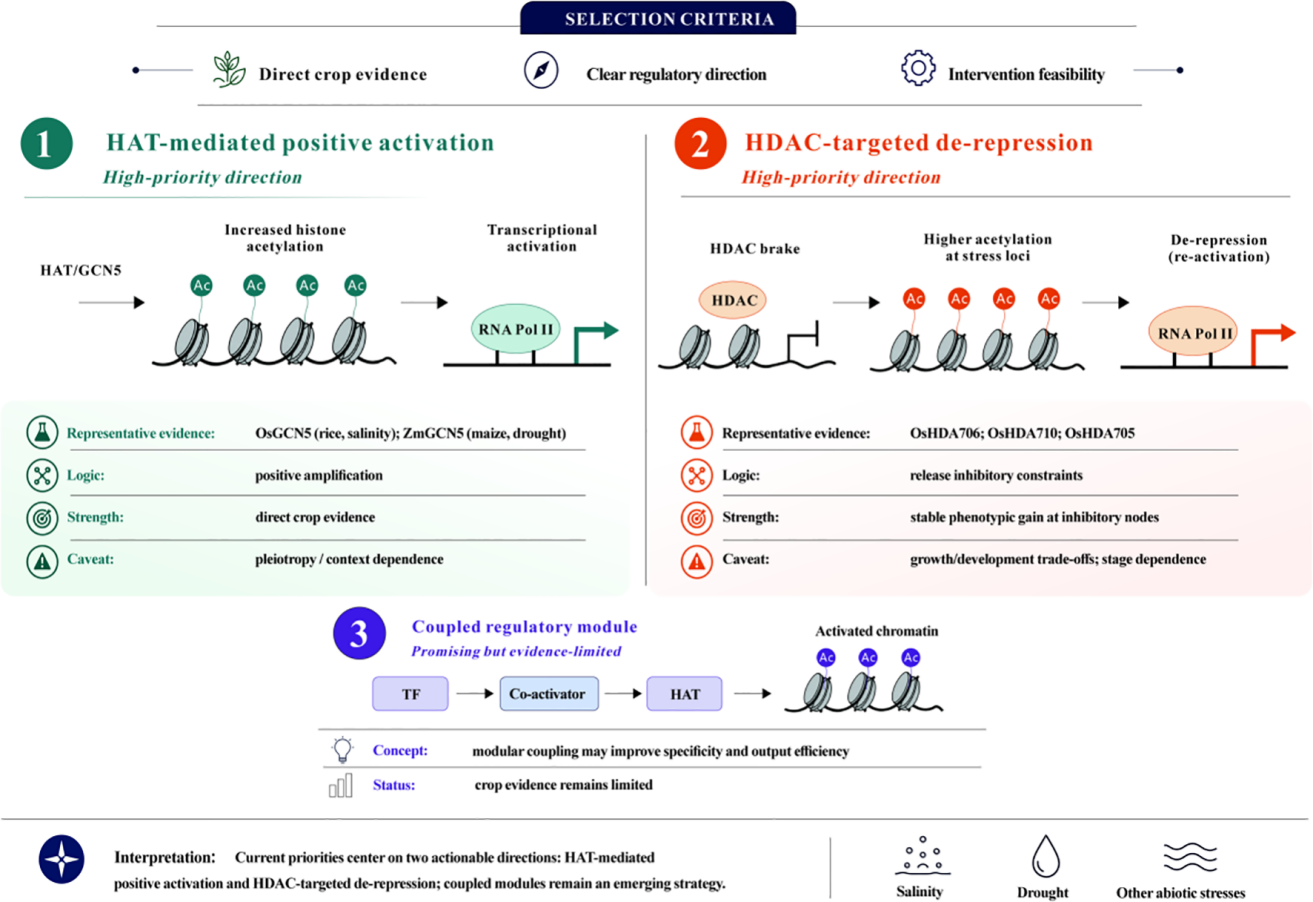
Prioritizing acetylation-based intervention strategies for crop stress-resilience improvement.

From the perspective of crop stress-resilience improvement, whether an acetylation-related regulator can serve as a priority target does not depend only on whether it participates in a single stress response. It also cannot be judged simply by the idea that “HATs are beneficial and HDACs are unfavorable.” Here, crop stress resilience includes tolerance to single stress events, faster secondary responses after priming, and adaptation to heterologous stresses through shared signaling, chromatin states, and metabolic priming. Therefore, priority target selection should consider direct crop evidence, clarity of regulatory direction, intervention feasibility, and possible side effects on growth and quality. Based on these criteria, acetylation-related crop improvement strategies can be roughly divided into three types: HAT-mediated positive activation, context-dependent HDAC-related regulation, and transcription factor–coactivator–HAT coupled modules (**Figure 8**).

Among the currently studied targets, GCN5-like HATs represent one of the clearest positive regulatory candidates. The rationale for targeting these factors is to enhance HAT-mediated histone acetylation, increase the transcriptional output of stress-responsive genes, and thereby improve plant adaptation to salinity, drought, heat, submergence, or other stresses. In rice, loss of OsGCN5 inhibits growth and weakens salt tolerance, whereas overexpression of OsGCN5 enhances seedling adaptation to salt stress. This suggests that GCN5-like HATs have relatively clear positive regulatory potential in cereal crops (Xue et al., 2024). Similarly, in maize, ZmSCE1a enhances the stability of ZmGCN5 and is associated with reduced drought-induced oxidative damage and enhanced drought stress responses, suggesting that GCN5-like HAT-associated modules may have stress-enhancing potential in cereals (Feng et al., 2024). However, this does not mean that all HAT overexpression is suitable as a direct application strategy. Because HAT-mediated acetylation often affects broad transcriptional programs, practical use should preferentially consider tissue-specific, stress-inducible, or moderate expression regulation, rather than simple constitutive strong expression.

This figure classifies acetylation-related intervention strategies based on direct crop evidence, clarity of regulatory direction, and intervention feasibility. HAT/GCN5-mediated positive activation currently has relatively clear crop evidence. HDAC-related strategies need to be distinguished according to target identity, including weakening HDAC-mediated repression of stress-tolerance-promoting genes or using HDACs to repress negative regulators. Transcription factor–coactivator–HAT coupled modules have high potential for specificity and output efficiency, but they remain a frontier direction with limited engineering validation. These strategies may provide candidate entry points for designing crop materials with stress priming, rapid reactivation, and potential cross-tolerance.

In contrast to HAT-mediated activation, HDAC-related targets must be evaluated according to target identity and regulatory outcome. One direction is to weaken HDAC-mediated repression of stress-responsive genes with positive roles in stress tolerance. For example, rice OsHDA710 participates in ABA-mediated salt-stress regulation. Loss of OsHDA710 increases acetylation levels in multiple salt-responsive gene regions and is accompanied by enhanced salt tolerance (Ullah et al., 2021). This suggests that, in specific contexts, moderately weakening the repressive role of some HDACs may benefit stress-resilience improvement. Another direction is to use HDACs to repress negative regulators. OsHDA706 can regulate H4K5ac/H4K8ac deacetylation at the OsPP2C49 locus, suppress a negative regulatory node in ABA signaling, and thereby enhance rice salt tolerance (Liu et al., 2023). Therefore, HDACs should not be simply viewed as “targets that need to be inhibited.” If the target is a stress-tolerance-promoting gene, weakening the corresponding HDAC may be beneficial. If the target is a negative regulator, that HDAC may instead be a positive regulatory node that needs to be enhanced, maintained, or precisely used.

In addition to single enzyme nodes, coupled regulatory modules also warrant consideration as basic units for future application design. The rice SUB1A–ADA2b–GCN5 module shows that transcription factors, coactivators, and HAT complexes can jointly integrate stress signal recognition, acetylation modification, and downstream transcriptional output, thereby enhancing submergence tolerance (Lin et al., 2023). Compared with single-gene targets, such coupled modules are more suitable for future epigenome editing, inducible expression, and synthetic biology design. They may also improve regulatory specificity and output efficiency. However, they should still be viewed as a promising but not yet fully validated direction. Their complexity is higher than that of a single HAT or HDAC target, because they involve the expression timing of multiple components, tissue specificity, interaction stability, and downstream network effects. Their practical application is therefore more difficult than that of single-enzyme targets.

Overall, the application value of acetylation-related targets should not be judged by the simple idea that “HATs are beneficial and HDACs are unfavorable.” Instead, it should be graded according to specific regulatory modules. GCN5-like HATs represent promising positive regulatory targets. Nodes such as OsHDA710 represent repression-weakening strategies. OsHDA706–OsPP2C49 suggests that some HDACs can play positive roles by suppressing negative regulators. SUB1A–ADA2b–GCN5 and similar coupled modules provide a design basis for future epigenome editing and synthetic regulation. These priority nodes provide evidence-based targets for small-molecule intervention, epigenome editing, and molecular design breeding. They also provide candidate entry points for designing crop materials with stress priming ability, rapid reactivation capacity, and potential cross-tolerance. However, before these nodes can truly enter crop improvement, they still need to be evaluated across multiple environments, multiple genetic backgrounds, and yield/quality-related side effects.

### 4.2 Small-molecule regulation: rapid and reversible, but highly context-dependent

Compared with gene editing and epigenome editing, small-molecule regulators provide a flexible, rapid, and reversible route to modulate acetylation networks. Existing studies have shown that exogenous compounds can alter histone acetylation states in plants within a relatively short time and induce remodeling of stress-related transcriptional programs, thereby improving plant adaptation to salinity, pathogen infection, drought, or other stresses to some extent (Kim et al., 2017; Xu et al., 2022). However, the application value of small-molecule regulation should not be judged only by whether it increases overall acetylation. Different compounds differ greatly in their mode of action, target specificity, effective concentration, treatment timing, and crop background. Therefore, evidence grading and context-specific evaluation are necessary. Overall, small-molecule regulation is more suitable as a tool for mechanistic validation or as an auxiliary measure in specific production scenarios. It should not be directly viewed as a broadly applicable field technology for stress tolerance.

Compounds such as TSA, SAHA, NaBT, and LBH-589 are usually classified as typical HDAC inhibitors. Their common feature is that they can rapidly increase histone acetylation levels by inhibiting HDAC activity and reshape part of the stress-responsive transcriptional program under controlled conditions. Rice blast-related studies provide an important warning: although NaBT, SAHA, LBH-589, and TSA can all affect histone acetylation levels to varying degrees, only 10 mM NaBT significantly enhances rice seedling resistance to blast disease (Xu et al., 2022). This shows that global changes in HAT/HDAC balance or overall increases in acetylation do not necessarily translate into stable beneficial disease-resistance phenotypes. In other words, the key to small-molecule regulation is not whether acetylation increases, but which gene loci or protein substrates are effectively regulated under a specific dose, tissue, developmental stage, and stress context.

Natural products and plant-derived small molecules may provide complementary chemical diversity for future screening of stress-regulatory compounds. However, direct evidence linking such compounds to HAT/HDAC activity, acetylation-state remodeling, or acetylation-mediated plant stress memory remains limited. Therefore, examples such as plant-derived extracts should be treated as sources of future screening inspiration rather than as established evidence for acetylation-based crop resilience (Dong et al., 2026).

In addition to direct HDAC inhibitors, some metabolism-related compounds may also affect stress adaptation through acetylation-related pathways. For example, acetate metabolism can participate in drought tolerance in *Arabidopsis* by influencing acetyl-CoA supply, histone H4 acetylation, and JA-related signaling priming (Kim et al., 2017). This type of evidence suggests that small-molecule regulation does not only include direct HDAC inhibition. It can also indirectly influence acetylation networks by changing metabolic substrates, acetyl donors, or hormone signaling states. Compared with direct HDAC inhibitors, this type of metabolism-coupled regulation may be closer to the plant’s own stress-adaptation logic. However, its pathway is also more complex and is easily affected by carbon metabolic status, developmental stage, and environmental conditions.

Therefore, small-molecule regulation is more suitable for two uses. First, it can serve as a mechanistic research tool to rapidly perturb acetylation states and test whether certain HAT/HDAC nodes, target genes, or non-histone substrates participate in specific stress responses. Second, in some cultivation scenarios, small-molecule treatment may serve as a short-term, reversible, and context-dependent auxiliary strategy to enhance seedling stress tolerance, induce defense states, or improve stress adaptation under particular environmental conditions. However, it should not currently be considered a general strategy that can replace genetic improvement. Treatment cost, effective dose, residue risk, field stability, differences in absorption and metabolism among crops, and potential side effects on yield and quality are all important limitations for field application.

Overall, the value of small-molecule intervention lies in its rapid and reversible nature, but its limitation also comes from its non-specificity and context dependence. Future research should shift from asking “whether overall acetylation is increased” to asking “which loci, which substrates, and which transcriptional modules are regulated under specific treatment conditions.” Time-series ChIP-seq, transcriptomics, acetyl-proteomics, and field phenotyping should be integrated to establish dose–target–phenotype relationships for small-molecule regulators. Only when the target is clear, the dose is safe, the phenotype is stable, and side effects on crop quality are controllable can small-molecule regulation move from an experimental tool toward an auxiliary stress-resilience strategy in specific production scenarios.

### 4.3 Epigenome editing and synthetic modules: frontier potential, but not yet mature field technology

Compared with traditional overexpression, knockout, or exogenous small-molecule treatment, epigenome editing and synthetic biology strategies better match the spatiotemporal specificity of acetylation regulation. Acetylation modification is highly dynamic, and its effect often depends on specific gene loci, tissue types, stress stages, and recovery processes. Therefore, a more targeted intervention idea is not to globally increase or decrease acetylation, but to guide HAT or HDAC activity to target gene loci or target regulatory modules under specific stress conditions, thereby achieving controllable epigenetic-state reprogramming.

CRISPR/dCas platforms provide a technical basis for this type of locus-specific regulation. By fusing catalytically inactive Cas proteins with transcriptional activation domains, HAT catalytic domains, or other epigenetic regulators, it is theoretically possible to alter the chromatin state of target promoters or enhancers without changing the DNA sequence (Moradpour and Abdulah, 2020; Cheng et al., 2024). In plants, dCas9-HAT targeting the *AREB1* promoter provides an exploratory technical route for enhancing the expression of stress-related genes and improving drought tolerance in *Arabidopsis* (Paixao et al., 2019). This case is directly relevant to acetylation regulation because the effector domain is a HAT module. Nevertheless, even in this example, promoter activation and stable acetylation-mediated stress memory should be distinguished. Increased gene expression and improved drought tolerance do not automatically demonstrate the formation of a persistent or reactivatable memory state.

CRISPRa activation of defense-related genes provides another useful but more indirect example. In tomato, CRISPRa-mediated activation of *SlPR-1* enhances defense-related gene expression and improves resistance to Clavibacter michiganensis subsp. michiganensis (García-Murillo et al., 2023). Similarly, dCas12a-SET targeting of *SlPAL2* increases *SlPAL2* expression, promotes lignin accumulation, and enhances tomato resistance to bacterial canker disease (Rivera-Toro et al., 2025). However, these examples mainly demonstrate the feasibility of targeted transcriptional activation or other active chromatin modifications in crop defense regulation. Unless the system uses a HAT effector domain or directly detects changes in acetylation marks such as H3K9ac or H3K27ac, they should not be interpreted as direct evidence of acetylation editing. Therefore, these cases are better viewed as proof-of-concept examples showing that stress- or defense-related loci can be programmably activated, rather than as direct evidence that acetylation editing can establish stress memory or cross-tolerance.

From an application-design perspective, combining a stress-inducible promoter with a HAT/HDAC fusion module represents a promising frontier strategy. The basic idea is to use promoters induced by salinity, drought, heat, or pathogens to locally activate HAT/HDAC-related regulatory modules when stress occurs, thereby enhancing the activation or release from repression of key stress-responsive genes. Under non-stress conditions, this design would minimize interference with normal growth and development. Compared with constitutive overexpression, this conditional regulation is more likely to reduce growth–defense trade-offs and better matches the dynamic, reversible, and time-dependent features of acetylation modification. A second application direction is to design synthetic acetylation modules based on mechanistic nodes discussed in earlier sections. For example, GCN5/ADA2b-related activation modules may serve as references for artificial transcriptional coactivation design; the OsHDA706–OsPP2C49 pathway may inspire modules that enhance stress signaling by suppressing ABA negative regulators; and the SUB1A–ADA2b–GCN5 module may provide a template for stress-inducible activation modules under submergence-responsive conditions (see Section 2.2; Lin et al., 2023; Xue et al., 2024). These examples indicate that synthetic module design should not focus only on single enzymes, but should consider the full regulatory logic, including upstream signals, chromatin effectors, target loci, and final adaptive outputs.

Natural product-based approaches may also provide complementary resources for future screening of compounds that modulate HAT activity, HDAC activity, histone acetylation, or non-histone acetylation states. However, current examples of plant-derived natural products, including compounds developed for eco-compatible crop protection, do not yet provide direct evidence for acetylation-mediated plant stress memory or cross-tolerance. Therefore, these examples should be interpreted as forward-looking screening resources rather than as established acetylation-based strategies for improving crop stress resilience (Suo et al., 2026).

Despite these possibilities, epigenome editing and synthetic acetylation modules are not yet mature field technologies. Their application in crops still faces several limitations. Delivery efficiency remains a major bottleneck, especially in many elite crop varieties. Tissue specificity and developmental timing are also critical, because sustained activation of stress-response pathways may impose growth or yield penalties. Off-target chromatin effects remain difficult to predict. In addition, it is still unclear whether edited acetylation states can be retained after stress release, reactivated during secondary stress, or maintained under fluctuating field environments (Cheng et al., 2024).

Another important challenge is how to balance stress protection with normal development. Stress-responsive acetylation is usually highly context-dependent. A given module may be beneficial under drought, salinity, submergence, or pathogen attack, but may be neutral or even detrimental under non-stress conditions. Therefore, future designs should not simply pursue constitutive activation. More feasible directions may involve stress-inducible promoters, synthetic sensor modules, tissue-specific expression systems, or reversible editing platforms, so that acetylation editing occurs only in the appropriate tissue, time window, and stress context.

Looking forward, the most promising direction may not be permanent activation of stress-responsive genes, but locus-specific and stress-inducible rewriting of acetylation states. If such acetylation editors can be combined with appropriate stress-responsive promoters or synthetic sensor modules, they could theoretically reinforce primed chromatin states at key stress-responsive loci. They may also help stabilize metabolic or hormonal states that favor rapid reactivation in response to subsequent homologous or heterologous stress. From this perspective, epigenome editing, synthetic acetylation modules, and natural product-based modulators provide complementary conceptual routes for translating acetylation-mediated stress-response mechanisms into programmable stress memory and cross-tolerance. However, this direction remains largely forward-looking and requires rigorous validation under recurrent stress, combined stress, and field-relevant crop environments.

### 4.4 Molecular design breeding: integrating genetic variation with epigenetic-state design

From the perspective of breeding, the ultimate value of acetylation-related research is not simply to propose new “epigenetic markers.” Rather, it is to provide interpretable, selectable, and combinable regulatory nodes for complex stress-resilience traits. Because acetylation states are dynamic, tissue-specific, and environmentally sensitive, they may not be suitable as stable genetic markers in the traditional sense. A more feasible route is to incorporate genetic variation, expression-regulatory variation, and functional haplotypes of HAT/HDAC genes and their target genes into molecular design breeding frameworks, while using acetylation states as an important functional layer for explaining genotype–phenotype relationships (Budhlakoti et al., 2022; Tyagi et al., 2024). Therefore, the main value of acetylation information in breeding is not as fixed markers for direct selection; rather, it helps explain which genetic variants establish favorable epigenetic regulatory states under stress.

Specifically, priority should be given to HAT/HDAC coding genes, regulatory complex components, and key downstream target genes with clear stress-tolerance or stress-resilience functions. Through GWAS, QTL, eQTL, and haplotype analyses in natural populations, germplasm resources, or breeding populations, functional variants associated with salt tolerance, drought tolerance, thermotolerance, submergence adaptation, or disease resistance can be identified (Budhlakoti et al., 2022; Tyagi et al., 2024). If certain variants are not only associated with stress-tolerance phenotypes, but also linked to HAT/HDAC expression under stress, acetylation levels at target genes, or the activation ability of downstream stress-resistance genes, these loci are more likely to become priority candidates in molecular design breeding. In other words, stable genetic variation can serve as the breeding handle, whereas acetylation states can serve as a mechanistic layer for understanding and optimizing the effects of these variants.

In crop application, functional haplotypes and regulatory variants may be easier to incorporate into breeding systems than dynamic acetylation marks themselves. For example, if promoter variation in a HAT or HDAC gene affects its stress-inducible expression strength and further changes acetylation and expression states of target stress-resistance genes, this type of variation may be developed as a molecular marker or gene-editing target. Similarly, if cis-element variation in the promoter region of certain target genes affects HAT/HDAC complex recruitment or stress-responsive acetylation dynamics, it may also provide an important entry point for improving stress-response thresholds and expression strength. Compared with directly selecting acetylation states, selecting these stable genetic variants is more compatible with conventional breeding and genomic selection systems.

However, acetylation-related nodes must pass strict filtering before they can truly enter molecular design breeding. First, the phenotypic effect of a candidate node needs to be repeatedly validated in multiple genetic backgrounds, rather than being effective only in a single variety or a single experimental condition. Second, enhanced stress resistance should not impose obvious costs on growth, yield, maturity, or quality. This is especially important in horticultural crops such as tomato, where SlHDA, SlHDT, and SlGCN5/SlHLS2-related nodes often affect ripening, quality, shelf life, disease resistance, and abiotic stress responses at the same time. Therefore, their breeding value cannot be evaluated only from the perspective of “enhanced stress resistance.” Third, candidate nodes must be assessed across multiple environments, years, and combined-stress conditions, because field stress is usually not a single, short-term, controllable stimulus, but a combination or alternation of multiple stresses.

Future molecular design breeding can use acetylation regulatory information at two levels. The first is the “genetic layer,” which uses functional haplotypes, eQTLs, promoter variants, or coding-region variants of HATs/HDACs and their target genes for marker-assisted selection, genomic selection, or precision editing. The second is the “epigenetic-state layer,” which uses induced by stress acetylation maps, time-series ChIP-seq, acetyl-proteomics, and transcriptomic data to explain why different genotypes show different response speeds, recovery capacities, and cross-adaptation potential under stress. Only by combining these two layers can acetylation research move from basic mechanistic discovery toward crop stress-resilience trait design.

Overall, acetylation regulation provides a new modular perspective for crop stress-resilience improvement. However, its application must be built on strict evaluation of evidence strength and crop context. HAT-mediated activation nodes, repression-weakening HDAC nodes, negative-regulator-suppressing HDAC nodes, reversible small-molecule intervention, epigenome editing, and molecular design breeding represent application routes with different levels of maturity and risk. The truly valuable direction in the future is not to broadly pursue global changes in acetylation levels, but to precisely design acetylation modules with clear regulatory logic and complete evidence chains around specific crops, specific stresses, and specific trait goals.

## 5 Conclusions and perspectives: unresolved questions and future directions

### 5.1 Current consensus and major bottlenecks

Overall, acetylation is increasingly recognized as an important regulatory layer in plant stress responses and may also contribute to the establishment of stress memory and cross-tolerance. Current studies show that histone acetylation participates in rapid plant responses to drought, salinity, heat, pathogen infection, and other environmental stimuli by regulating chromatin accessibility and the transcriptional activity of stress-related genes (Hu et al., 2019; Jiang et al., 2020; Kumar et al., 2021; Wang et al., 2024). At the same time, non-histone lysine acetylation is increasingly understood to participate in metabolic reprogramming, protein stability control, photosynthetic regulation, and immune signaling, extending the functional boundary of acetylation beyond the traditional framework of “histone modification” (Xia et al., 2022; Jin et al., 2024). In other words, acetylation in plants should no longer be viewed simply as an epigenetic modification that locally regulates selected genes. Rather, it is better understood as a regulatory hub linking environmental signals, metabolic status, and phenotypic plasticity (Hu et al., 2019; Lu et al., 2023). Examples discussed above, such as OsGCN5 and OsHDA706 in rice and TaHAG1 in wheat, provide relatively clear crop evidence connecting acetylation regulators with stress-related phenotypes (Zheng et al., 2021; Liu et al., 2023; Xue et al., 2024; Yang et al., 2024).

Despite this progress, the field still faces several common bottlenecks. First, the systematic characterization of non-histone acetylation substrates remains insufficient. Plant acetylome studies have developed rapidly in recent years, and increasing numbers of non-histone acetylation events have been identified (Xia et al., 2022; Jin et al., 2024). However, relatively few studies have established a complete causal chain from acetylation site to protein function, signaling pathway, and stress phenotype. This problem is particularly prominent in crops. Many identified acetylated proteins still remain at the catalog-description stage, and it remains unclear whether specific modification sites truly determine protein activity, stability, interaction capacity, or stress-resilience phenotypes. Second, the temporal and spatial resolution of acetylation dynamics remains limited. Most current studies rely on ChIP, immunoblotting, or endpoint proteomics to capture acetylation states. These methods can reveal modification differences at specific time points, but they are less able to resolve the continuous dynamics of acetylation during stress onset, recovery, memory maintenance, and recurrent stimulation. They also provide limited information on tissue-, cell-, or subcellular-level heterogeneity (Oberkofler et al., 2021; Xia et al., 2022; Kappel et al., 2023). Third, modification networks under combined-stress conditions remain difficult to disentangle. In natural environments, plants often experience multiple stresses at the same time, including temperature fluctuations, water deficit, salinity, and pathogen infection. Because acetylation is also extensively intertwined with hormone signaling, ROS dynamics, energy metabolism, and other epigenetic modifications, the field still lacks a sufficiently clear analytical framework to distinguish shared stress-response acetylation events from stress-specific or tissue-specific regulatory events (Zandalinas et al., 2024; Jiang et al., 2025).

In addition, there remains a clear translational bottleneck between mechanistic discovery and crop application. Many acetylation-related stress studies are still mainly based on controlled environments, single genetic backgrounds, or single-stress treatments. Field stress, however, is usually characterized by multiple environments, multiple years, multiple genotypes, and alternating or combined stresses. Therefore, whether candidate HAT/HDAC nodes or acetylation regulatory modules have stable application value still needs to be validated in target crops across multiple genetic backgrounds, environments, and combined-stress conditions. It is especially important to distinguish acetylation events that have been causally shown to contribute to stress phenotypes from those that are currently only associated with stress-responsive states. Only the former are more likely to become reliable targets for crop improvement (Budhlakoti et al., 2022; Tyagi et al., 2024).

### 5.2 Frontier directions and future research priorities

In response to these bottlenecks, future plant acetylation research should move further from “candidate-site validation” toward “network-level analysis.” In this direction, spatially informed, high-resolution plant acetyl-proteomics will be foundational. Technical routes based on acetyl-lysine antibody enrichment combined with liquid chromatography–tandem mass spectrometry have already enabled large-scale identification of lysine acetylation sites in plants, providing an important basis for systematic analysis of acetylation networks (Xia et al., 2022; Jin et al., 2024). However, plant tissues are highly heterogeneous, low-abundance modification sites are difficult to enrich, and induced by stress acetylation changes often show rapid dynamics. These features indicate that this technical system still has considerable room for improvement. Future advances in sampling resolution, quantitative accuracy, subcellular localization, and tissue-specific analysis will help identify key acetylation events that truly participate in stress responses, stress memory, and crop stress-resilience phenotypes.

A second important direction is to construct a time-resolved acetylation atlas centered on stress memory. Existing studies on heat stress memory and plant epigenetic memory indicate that memory formation is not simply the retention of a single modification state. Instead, it is a continuous process involving initial stress stimulation, recovery-phase maintenance, recurrent stress, and rapid reactivation (Lämke and Bäurle, 2017; Oberkofler et al., 2021; Charng et al., 2023; Kappel et al., 2023). Therefore, acetylome or transcriptome data from a single time point are not sufficient to distinguish ordinary short-term stress responses from acetylation events that genuinely participate in memory maintenance. In the future, time-resolved acetylome profiling should be integrated with chromatin-state analysis and transcriptomics to construct multi-phase dynamic maps covering “initial stress—recovery—memory maintenance—recurrent stress—reactivation.” Such maps will help identify which acetylation events are retained after stress release, which are reset, and which are rapidly restored upon recurrent stress, thereby clarifying the key temporal windows and molecular basis of stress memory and cross-tolerance.

Third, interactions between the rhizosphere microbiome and host acetylation deserve attention as a longer-term frontier direction. In recent years, plant microbiome research and epigenetics have shown an increasingly clear trend of convergence, and related studies have begun to discuss the concept of “microbiome–epigenome interaction” (Vannier et al., 2015; Vílchez et al., 2024). Available evidence suggests that rhizosphere microorganisms and their metabolites may indirectly alter host chromatin states and response-gene expression by influencing plant hormone status, redox balance, carbon metabolism, and metabolic substrate supply (Chen et al., 2022; Doddavarapu et al., 2024). Conversely, changes in host epigenetic states may also affect root exudate composition and microbial community structure. However, studies directly focusing on plant acetylation–rhizosphere interactions remain limited. Therefore, this direction is more suitable as a future frontier at the systems-ecology level and should not be regarded too early as a mature based on acetylation regulation stress-resilience mechanism. Future studies could focus on whether testable causal links exist among microbial metabolites, acetate/acetyl-CoA supply, hormone signaling, and root epigenetic states.

Fourth, the possible contribution of acetylation to parental stress effects, transgenerational stress memory, and crop domestication warrants long-term investigation. Under certain conditions, parental environmental stress may influence phenotypic plasticity and stress responsiveness in the immediate offspring, which first represents an intergenerational effect; only when such environmentally induced regulatory states persist in later generations that are no longer exposed to the original stress can they be more strictly considered transgenerational inheritance (Lämke and Bäurle, 2017). However, uncertainties remain regarding the duration and heritability of plant stress memory and whether relevant epigenetic marks can stably escape reprogramming (Crisp et al., 2016; Oberkofler et al., 2021; Cao and Chen, 2024). In particular, there is still no consensus on which epigenetic marks can stably escape reprogramming, how long they can be maintained across generations, or how much acetylation contributes to these processes (Tricker, 2015; Cao and Chen, 2024). Therefore, discussions of “transgenerational acetylation memory” should remain cautious. A more appropriate framing is to explore whether and how acetylation may participate in parental stress effects and transgenerational stress memory. The value of this direction lies not only in explaining how plants “remember” environmental experience, but also in exploring whether environmentally induced epigenetic variation could serve as an additional source of variation in crop evolution, domestication, and modern breeding.

Overall, near-term priorities should include causal validation of non-histone acetylation substrates, high-resolution acetylome analysis, and construction of time-resolved stress-memory acetylation maps. Microbiome-associated acetylation regulation and the possible role of acetylation in transgenerational stress memory are better viewed as longer-term frontiers at the systems and evolutionary scales. This distinction in priority can help prevent future directions from becoming too scattered and can help acetylation research move more effectively from correlation-based description toward causal mechanistic analysis.

### 5.3 Concluding remarks

Taken together, the function of acetylation in plants should no longer be understood simply as a transcriptional “on/off” switch. Instead, it should be viewed as an important regulatory hub integrating environmental signals, metabolic status, chromatin plasticity, and phenotypic responses (Hu et al., 2019; Lu et al., 2023; Wang et al., 2024). Acetylation not only participates in rapid responses to ongoing stress, but may also contribute to stress memory and cross-tolerance through three interconnected processes. It can facilitate chromatin reactivation at stress-responsive loci, support metabolic priming, and remodel the functional states of non-histone proteins (Lämke and Bäurle, 2017; Oberkofler et al., 2021; Jin et al., 2024). It may also be associated with longer-term adaptive states; however, whether acetylation directly contributes to parental stress effects or acts as a stable carrier of transgenerational information still requires more rigorous multigenerational evidence. Overall, acetylation is best supported as a dynamic regulator of stress responses and somatic stress memory, whereas its roles in long-term cross-tolerance and transgenerational inheritance remain promising but less directly validated. Evidence for acetylation in general stress responses is relatively strong, whereas its causal roles in long-term stress memory, cross-tolerance, and transgenerational adaptation still require further confirmation through time-resolved multi-omics combined with genetic perturbation of HAT/HDAC modules, as well as locus-specific acetylation editing, acetylation-site mutagenesis, or genetic complementation experiments (Kappel et al., 2023; Cao and Chen, 2024).

From an applied perspective, acetylation research is expanding crop stress-resilience improvement beyond traditional genotype selection toward epigenetic-state-informed design. The point is not to replace stable genetic variation with dynamic acetylation states, but to build a clearer explanatory framework linking genetic variation, epigenetic states, and stress phenotypes. With continued advances in high-resolution acetylomics and time-resolved epigenetic mapping, future studies will be better able to systematically resolve acetylation dynamics during stress, recovery, and recurrent stress. At the same time, advances in plant epigenome-editing platforms may help translate these mechanistic insights into programmable crop stress-resilience design strategies (Paixao et al., 2019; Xia et al., 2022; Kappel et al., 2023; Wang et al., 2024). The key question is not whether acetylation increases globally, but which acetylation nodes can be translated into beneficial phenotypes in specific crops, tissues, stress stages, and environments.

## Data Availability

Publicly available datasets were analyzed in this study. These data can be found at Ensembl Plants: https://plants.ensembl.org/.
